# Sensing a rainbow of colors: algal photoreceptors

**DOI:** 10.3389/fpls.2025.1684559

**Published:** 2025-10-14

**Authors:** Armin Hallmann

**Affiliations:** Department of Cellular and Developmental Biology of Plants, University of Bielefeld, Bielefeld, Germany

**Keywords:** algae, channelrhodopsins, photobiology, light perception, light sensing, photoreceptors, photosensors, optogenetics

## Abstract

Algae inhabit diverse environments with highly variable light conditions, making light sensing essential for survival and ecological success. This review explores the remarkable diversity of algal photoreceptors, enabling detection and response to a broad spectrum of sunlight, from ultraviolet to far-red wavelengths. Algae utilize various light-sensitive proteins—including flavin-based receptors (phototropins, cryptochromes, aureochromes, BLUF proteins), retinal-based rhodopsins, tetrapyrrole-based phytochromes, hybrid neochromes, and UV-B photoreceptors — to sense and integrate both light quality and quantity. The evolution of these photoreceptors has been shaped by endosymbiotic events, gene duplication, and domain fusion, equipping algae with robust mechanisms for environmental adaptation. Advances in genomics and transcriptomics have revealed many novel algal photoreceptors, some of which are being harnessed as optogenetic tools in biomedical research. Channelrhodopsins from green algae, for example, have revolutionized neuroscience by enabling precise, light-controlled manipulation of neuronal activity. The ongoing discovery and engineering of algal photoreceptors continue to expand the molecular toolkit for both basic research and practical applications. In summary, algal photoreceptors exemplify evolutionary innovation in adapting to diverse light environments and underpin numerous physiological processes critical for algal survival. Study and exploitation of these proteins offer profound insights into light perception, signaling, and technological applications, particularly in the rapidly growing field of optogenetics.

## 1 Introduction

**Figure 1:**
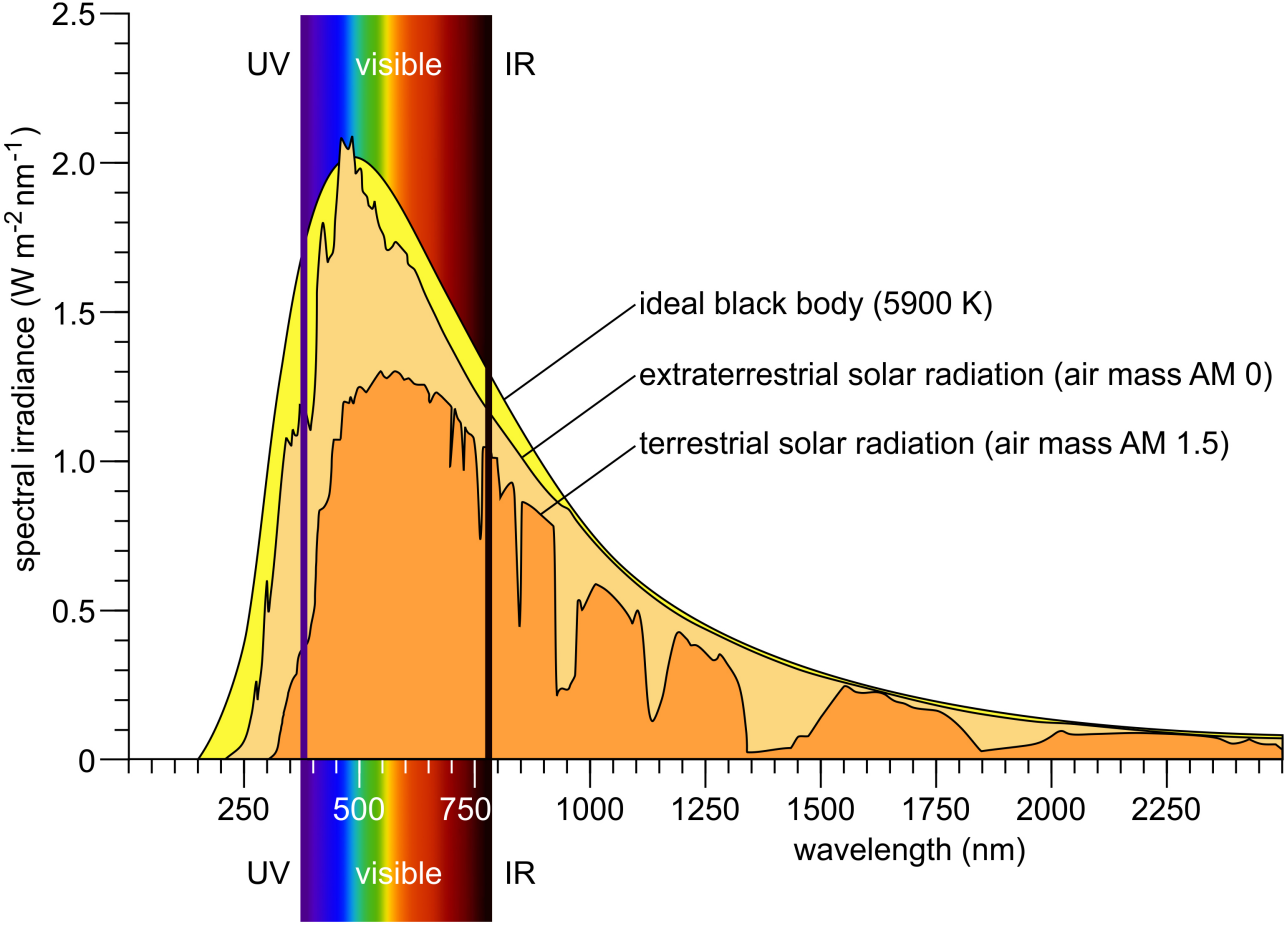
Solar irradiance spectrum. The surface temperature of the Sun is approximately 5900 K; therefore, a black-body radiation curve at this temperature serves as a theoretical model for the Sun’s emitted light. This model is compared to the solar irradiance spectrum measured outside Earth’s atmosphere (extraterrestrial solar radiation; air mass AM 0) and to the spectrum observed at Earth’s surface when the Sun is 48.2° above the horizon—typical for midday conditions at many locations (terrestrial solar radiation; air mass AM 1.5). Redrawn and modified after Iqbal (1983); Kirk (1994); Goswami et al. (1999); Wang et al. (2021); Leppäranta (2023).

**Figure 2:**
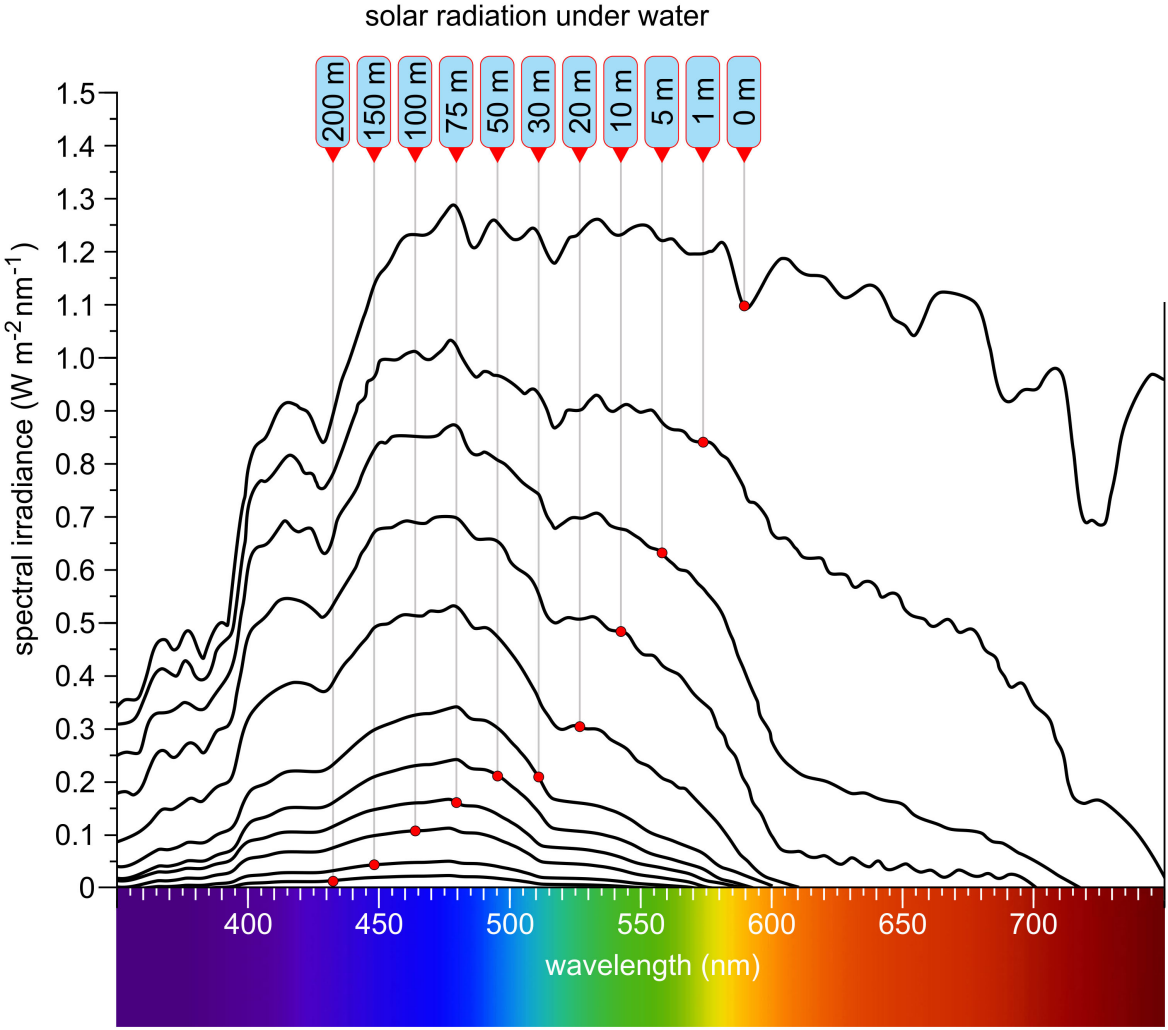
Solar radiation at different depths in clear water. Labels are arranged with the shallowest depth on the right and the greatest depth on the left. As depth increases, longer wavelengths are progressively attenuated, and the overall light intensity diminishes. Far-red and red wavelengths are absorbed first, followed by orange and yellow. Green light penetrates deeper, while blue and violet-blue wavelengths reach the greatest depths—up to approximately 200 meters. At this depth, typically only about 0.1% to 0.5% of the surface light intensity remains. Redrawn and modified after Smith and Baker (1981); Muaddi and Jamal (1991); Kirk (1994); Tedetti and Sempere (2006); Simpson et al. (2014).

The Sun, despite being 150 million kilometers from Earth, is the driving force behind all life. Its emitted continuous spectrum ranges from X-rays through ultraviolet, visible, and infrared, with peak intensity in the blue-green (approximately 500 nm) (**Figure 1**). Only part of the extraterrestrial solar radiation reaches Earth’s surface; the atmosphere absorbs much of the X-ray and ultraviolet radiation and modifies the spectrum with thousands of absorption lines (Fraunhofer lines), reflecting both solar and atmospheric composition. Solar radiation relevant for algae and plants can be divided into ultraviolet (UV, 100–380 nm), visible (approximately 380–780 nm), and infrared (IR, >780 nm). Photosynthetically active radiation (PAR) spans approximately 400–700 nm, largely overlapping with human-visible light. Blue-violet (400–500 nm), green (500–575 nm), yellow-orange (575–620 nm), red (620–700 nm), and far-red (700–780 nm) constitute the major color bands, with IR subdivided further (Endres and Breit, 2009). When sunlight enters water—the main habitat of algae—the spectrum changes significantly (Smith and Baker, 1981; Muaddi and Jamal, 1991; Kirk, 1994; Tedetti and Sempere, 2006; Simpson et al., 2014; Mat et al., 2024): UVB is strongly attenuated, UVA less so. At 50 cm water depth, 85% of UVA remains, whereas only 60% of UVB does. Visible light above 600 nm is rapidly attenuated with depth, and below 10 m, virtually no red light remains (**Figure 2**) (Kirk, 1994). Below 50 m, only violet-blue, blue, and blue-green light persist, and at 200 m only traces of violet-blue and blue light remain (**Figure 2**).

**Figure 3:**
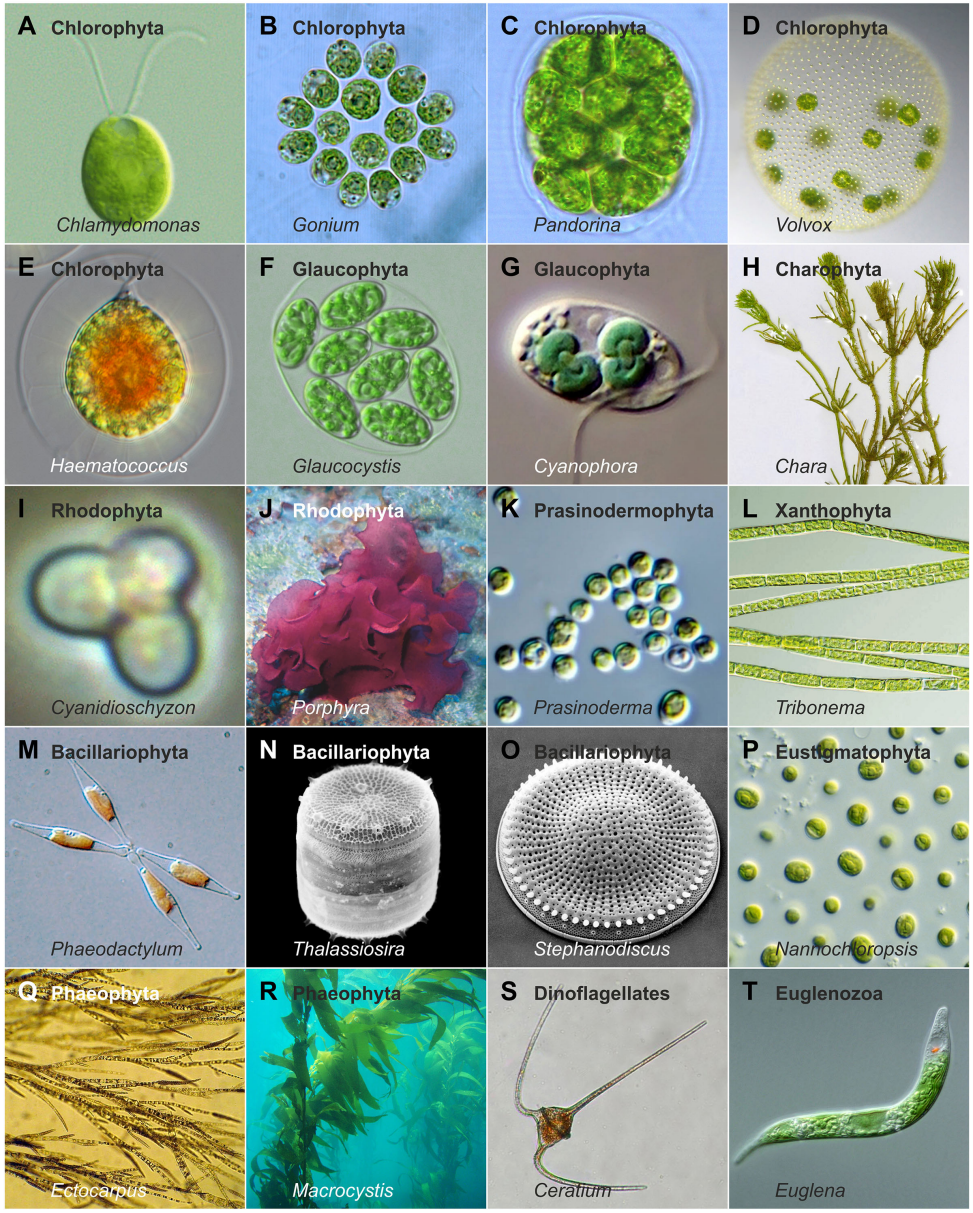
Example algal species illustrating morphological and ecological diversity. A selection of algal species illustrating diversity in cell number, structural complexity, size, motility or attachment strategies, pigmentation, habitat, and phylogenetic position. Photos by Alessandra de Martino and Chris Bowler (*Phaeodactylum*), Christian Fischer (*Chara*), Claire Fackler (*Macrocystis*), CSIRO (*Nannochloropsis*), djpmapfer (*Euglena*), Frank E. Round (*Stephanodiscus*), Keisotyo (*Ceratium*), Li and colleagues (Li et al., 2020) (*Prasinoderma*), Neon (*Cyanidioschyzon*), Nils Kröger (*Thalassiosira*), Sarka Martinez (*Ectocarpus*), Wiedehopf20 (*Haematococcus*), Wolfgang Bettighofer (*Cyanophora*), Y. Tsukii (*Glaucocystis*), and own work (Hallmann, 2011; 2015;Kianianmomeni and Hallmann, 2016; Hallmann, 2020, 2024).

Despite the wide variety of light conditions, algae have successfully colonized virtually all aquatic environments and are also found in many other settings. The habitats they occupy differ greatly not only in light availability, intensity, and spectral quality, but also in water properties such as salinity, pH, turbidity, depth (**Figure 2**), temperature, nutrient content, and other ecological factors. All these parameters together influence the growth and light absorption of the algae inhabiting these environments. In this review, the term ‘algae’ refers specifically to photosynthetic eukaryotic algae, excluding prokaryotic phototrophs such as cyanobacteria (often referred to as ‘blue-green algae’ in older literature). The diversity of eukaryotic algae encountered across habitats could hardly be greater: they differ in color, motility, cell number, phylogenetic position, and structural complexity, ranging from unicellular to colonial and multicellular forms (Graham and Wilcox, 2000; Barsanti and Gualtieri, 2014), with the smallest species (*Ostreococcus tauri*, Prasinodermophyta) under 1 µm (Courties et al., 1994) and the largest species (*Macrocystis pyrifera*, Phaeophyta) (**Figure 3**) exceeding 60 meters (Hallmann, 2007). Some multicellular algae consist exclusively of cell clusters with similar cells or simple filaments, while others are composed of tissues (**Figure 3**). In many species, the evolution of multicellularity has been accompanied by the differentiation of specialized cell types (Graham and Wilcox, 2000; Guiry, 2012; Barsanti and Gualtieri, 2014).

**Figure 4:**
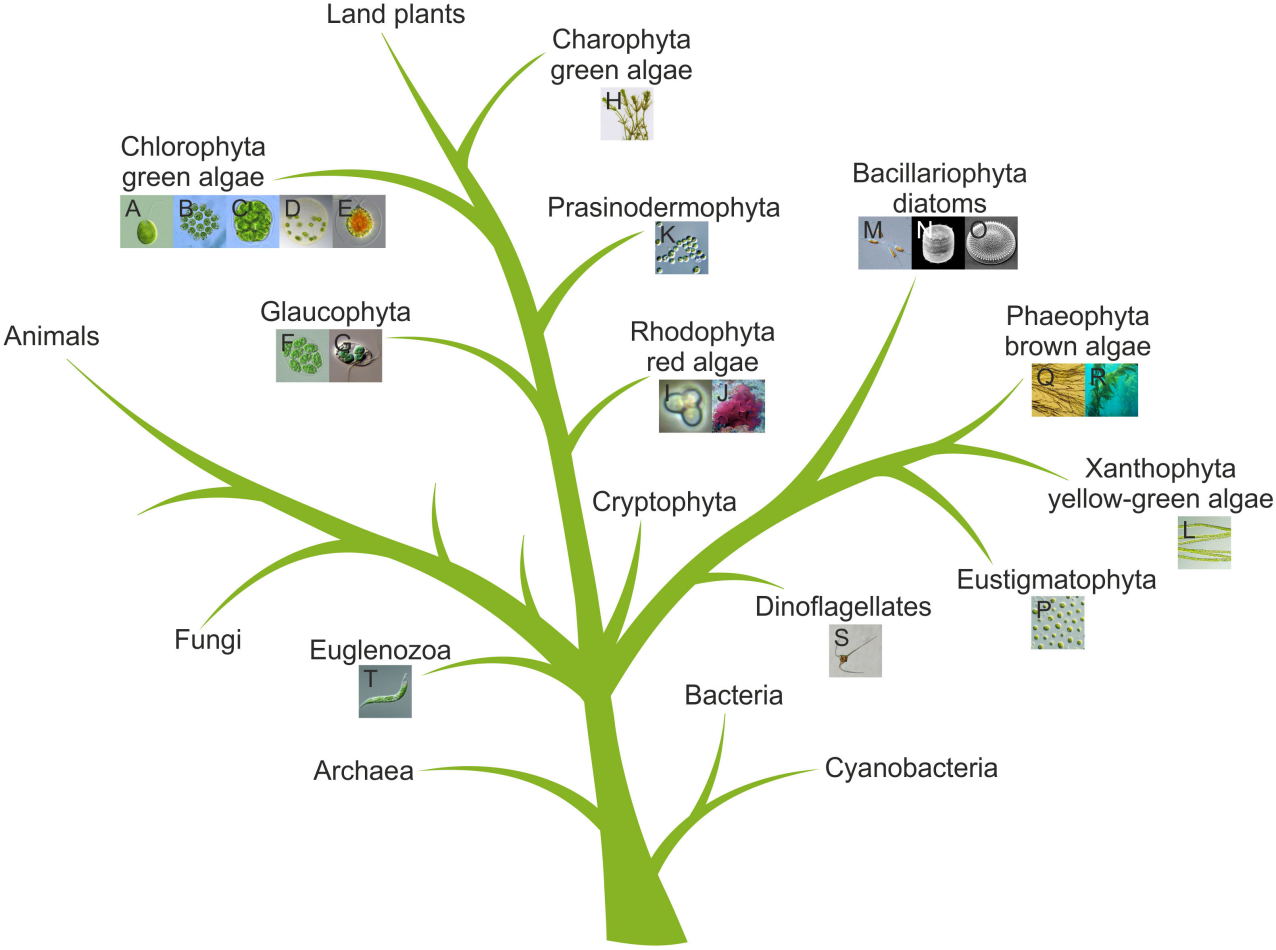
Simplified tree of life highlighting algae. The tree is based on previous phylogenetic analyses (Kranz et al., 1995; Baldauf, 2003; Maddison and Schulz, 2007; Prochnik et al., 2010; Hallmann, 2011; Kianianmomeni and Hallmann, 2014). Algal species from **Figure 3** are shown as thumbnails and are assigned to their respective groups. The letters in the thumbnails correspond to those in **Figure 3**.

Given the vast diversity of algae—currently about 177,000 documented species, with estimates reaching up to one million (Guiry, 2012; Raven and Giordano, 2014)—and their ecological importance, understanding algal (photo)biology has become a major focus of research. The principal algal groups and their phylogenetic relationships are illustrated in a simplified tree of life (**Figure 4**). To unravel fundamental molecular and cellular principles, a number of algal model species have been established, including *Chlamydomonas reinhardtii* (Chlorophyta) (**Figure 3**), *Volvox carteri* (Chlorophyta) (**Figure 3**), *Cyanidioschyzon merolae* (Rhodophyta) (**Figure 3**), *Phaeodactylum tricornutum* (Bacillariophyta) (**Figure 3**), *Vaucheria frigida* (Xanthophyta), *Mougeotia scalaris* (Charophyta), *Ectocarpus siliculosus* (Phaeophyta) (**Figure 3**) and others (Kianianmomeni and Hallmann, 2014).

Previous reviews have addressed various aspects of photoreceptors (Hegemann et al., 2001; Kateriya et al., 2004; Hegemann, 2008; Kami et al., 2010; Möglich et al., 2010; Kianianmomeni and Hallmann, 2014; Parihar et al., 2016; Duanmu et al., 2017; Kottke et al., 2017, 2018; Sasso et al., 2018; Petersen et al., 2021; Shankar et al., 2022; Vierock and Hegemann, 2023). Building on these and further foundations, this review first outlines the diversity of light environments and habitats that algae occupy and describes the fundamental physiological processes in algae that are regulated by light. The evolutionary origins and habitat adaptations of algal photoreceptors are then discussed, followed by a brief overview of the scientific history of their discovery. The main classes of algal photoreceptors—including their molecular structures, chromophores, and mechanisms of action—are reviewed in detail. Recent advances in algal genomics and transcriptomics as sources for the discovery of new light-sensitive proteins are highlighted. Finally, current and emerging applications of algal photoreceptors in optogenetics are discussed, and an outlook on future directions in this rapidly evolving field is provided.

## 2 Light-dependent processes in algae

Light serves algae not only as an energy source for photosynthesis but also as an environmental cue regulating a multitude of physiological and developmental processes. Light-harvesting pigments and complexes capture photons to drive photosynthesis, which converts solar energy into chemical energy for growth and energy storage (Falkowski and Raven, 2007; Croce and Van Amerongen, 2014). However, light is also sensed through specialized photoreceptors, which detect changes in light intensity and quality.

Upon light detection, various signal transduction cascades are activated. Light-dependent ion channels initiate ion currents that alter membrane potential and activate downstream signaling pathways. Key second messenger systems include cyclic nucleotides (cGMP, cAMP), calcium ions, and reactive oxygen species (ROS). For example, changes in cGMP/cAMP modulate enzymes, rapid Ca²^+^ fluxes influence flagellar movement and gene expression, and high light intensities generate ROS, which act as signals for stress responses or the induction of sexual reproduction.

Light coordinates gene expression and protein turnover in algae, aligning photosynthetic capacity and metabolism with prevailing light conditions. It acts across all regulatory layers—from chromatin organization and transcription to translation, post-translational modifications, and metabolism. In doing so, light shapes numerous physiological responses, including:

– Photosynthesis and chloroplast adaptation: Algae adjust their photosynthetic machinery and pigment composition in response to light quality and quantity, optimizing energy capture while preventing photodamage (Kirk and Kirk, 1985; Grossman et al., 2004; Falkowski and Raven, 2007; Kupper et al., 2009; Ozawa et al., 2009; Roy et al., 2011; Frank and Cogdell, 2012; Nickelsen and Rengstl, 2013; Wada, 2013; Croce and Van Amerongen, 2014; Maltsev et al., 2021).
– Photoprotection: Protective mechanisms, including non-photochemical quenching, antioxidant production, and repair of photodamaged components, are activated by light (Demmig-Adams and Adams, 1996; Niyogi, 1999; Allorent and Petroutsos, 2017; Vecchi et al., 2020).
– Photobehavior and phototaxis: Algae can move toward or away from light sources, optimizing their position for growth or protection (Lenci and Colombetti, 1978; Foster and Smyth, 1980; Kreimer, 1994; Sgarbossa et al., 2002; Harris et al., 2009; Sasso et al., 2018; Wakabayashi et al., 2021; Seth et al., 2022; Leptos et al., 2023).
– Phototropism: Some filamentous and other multicellular algae reorient growth in response to directional light (Rico and Guiry, 1996; Takahashi and Mikami, 2016; 2019).
– Circadian rhythms: Endogenous biological clocks synchronized by light regulate daily cycles of cell division, metabolism, and behavior (Mittag, 2001; Werner, 2002; Mittag et al., 2005; Matsuo et al., 2008; Matsuo and Ishiura, 2010; Schulze et al., 2010; Matsuo and Ishiura, 2011; Bouget et al., 2014; Galvão and Fankhauser, 2015; Noordally and Millar, 2015; Kuhlman et al., 2018; Farre, 2020; Matsuo et al., 2020).
– Development and morphogenesis: Light influences developmental pathways, including multicellular differentiation and photomorphogenic responses (Dring and Lüning, 1983; Kirk and Kirk, 1985; Dring, 1988; Lüning, 1990; Agarwal et al., 2023).
– Cell cycle and sexual reproduction: Light modulates the timing of cell division, the induction of sexual cycles, and gamete formation (Craigie and Cavalier-Smith, 1982; Donnan and John, 1983; Weissig and Beck, 1991; Pan et al., 1997; Saito et al., 1998; Zachleder et al., 2002; Huang and Beck, 2003; Oldenhof et al., 2004a, 2004; Goodenough et al., 2007; Mouget et al., 2009; Bisova and Zachleder, 2014; Cross and Umen, 2015; Müller et al., 2017; Zou et al., 2017; Permann et al., 2022).
– Pigment production: Light regulates the synthesis of photosynthetic and protective pigments (Begum et al., 2016; Lee et al., 2018; Mutschlechner et al., 2022; Zarekarizi et al., 2023).

Most physiological responses involve the integration of multiple photoreceptors (Kami et al., 2010). Moreover, light signals are often integrated with other environmental factors such as temperature, pH, salinity, nutrient availability, and biotic interactions to trigger specific adaptive responses.

## 3 Evolutionary origins and habitat adaptations of algal photoreceptors

For light to affect algal cells, it must first be absorbed by biomolecules. Light-harvesting pigments collect photons for photosynthesis, while sensory photoreceptors provide information about the environment, enabling cells to regulate growth, development, and stress responses. The interplay between light perception and downstream physiological reactions is critical for survival (Kirk and Kirk, 1985; Huang and Beck, 2003; Grossman et al., 2004). The algal light-sensing system must detect not only light intensity, but also its spectral composition.

Photoreceptors are evolutionarily ancient. Light-sensitive proteins already existed in early prokaryotes, where microbial rhodopsins such as bacteriorhodopsin acted as light-driven proton pumps for energy generation (Spudich et al., 2000; Ernst et al., 2014; Gushchin and Gordeliy, 2018). True photoreceptors, in contrast, evolved as signaling proteins that couple chromophore absorption to downstream regulatory responses (Möglich et al., 2010; Losi and Gärtner, 2012). Plastids originated from the primary endosymbiosis with a cyanobacterium-like ancestor (Keeling, 2010; Archibald, 2015; Ponce-Toledo et al., 2017). This event not only established photosynthesis in the host cell but was also accompanied by extensive endosymbiotic gene transfer from the endosymbiont to the host nucleus (Timmis et al., 2004; Archibald, 2015). Subsequent secondary (e.g., brown algae, diatoms) and tertiary endosymbioses (e.g., in some dinoflagellates) introduced plastids into additional eukaryotic lineages and contributed further genes to their nuclear genomes (Keeling, 2013; Dorrell and Howe, 2015).

The evolutionary trajectories of individual photoreceptor families remain complex and in several cases unresolved. While some photoreceptor types may trace back to cyanobacterial genes, others appear to have arisen independently or to have been shaped by convergent evolution, horizontal gene transfer (HGT), or lineage-specific innovation (Losi and Gärtner, 2012; Li et al., 2015a, 2015; Li and Mathews, 2016; Rockwell and Lagarias, 2020). Thus, the algal photoreceptor repertoire reflects a mosaic evolutionary history rather than a single ancestral inheritance.

Today, eukaryotic algae occupy a vast array of habitats: freshwater (rivers, lakes, ponds), wetlands, marine (coastal, open ocean, deep-sea, estuaries), and terrestrial (soils, rocks, tree bark). Some algae are airborne, colonize extreme sites (e.g., snow, hot springs, hypersaline lakes), or inhabit artificial environments (e.g., irrigation systems, buildings) (Raven and Giordano, 2014).

Light availability and spectral quality vary dramatically between habitats, particularly with water depth (**Figure 2**). Shallow waters are rich in red and blue light, but at greater depths only blue and violet wavelengths remain, and light intensity drops sharply. Also hypersaline waters lead to an altered light spectrum. Terrestrial algae, especially those on snow or ice, must withstand high-intensity UV and blue light. Low-light environments, such as those found in deeper water columns or densely shaded habitats, pose a particular challenge. Moreover, incident light is frequently spectrally filtered by overlying vegetation (e.g., leaves) or other algal biomass.

Adaptation to such variable light conditions has driven diversification of both light-harvesting pigments and photoreceptors, enabling algae to exploit a wide range of ecological niches.

## 4 A brief look at the scientific history of algal photoreceptors

Although light perception and response mechanisms have existed in algae for millions of years, scientific understanding of these processes is relatively recent. In the 19th century, Andrei S. Faminzyn observed that unicellular algae like *Chlamydomonas* (Chlorophyta, **Figures 3**, **4**) and *Euglena* (Euglenozoa, **Figures 3**, **4**) could move toward or away from light sources (Faminzin, 1866)—a phenomenon now known as phototaxis and an early indication of light sensitivity in algae. Early studies also identified carotenoids as algal pigments, and foundational research by Jan Ingenhousz and Robert Emerson established that light functions not only as an energy source but also as a regulatory signal (Emerson, 1957; Govindjee, 1999).

The mid-20th century saw the elucidation of photosynthesis mechanisms by researchers like Hill, Calvin, Benson, and Bassham (Jahn, 2004; Eberhard et al., 2008). In the 1950s–1970s, the discovery of blue light sensitivity in algae led to the hypothesis of specialized blue light photoreceptors. This period also saw the identification of flavin-based blue light receptors in algae, and the association of the algal eyespot with light-sensing pigments such as carotenoids (Briggs, 2006; Kreimer, 2009; Losi and Gärtner, 2011).

Subsequent decades brought the discovery of cryptochromes and phototropins in both higher plants and algae (Liscum and Briggs, 1995; Briggs et al., 2001; Chory, 2010; Yu et al., 2010; Beel et al., 2013; Christie et al., 2015). In the 1970s, rhodopsins—originally identified as visual pigments in animals—were found as bacteriorhodopsin in microorganisms, i.e. in halobacteria (Archaea, **Figure 4**) (Oesterhelt and Stoeckenius, 1971; 1973; Kayushin and Skulachev, 1974). In the early 2000s, microbial-type rhodopsins were identified in green algae such as *Chlamydomonas reinhardtii*, where they function as light-gated ion channels (channelrhodopsins) (Nagel et al., 2002; Sineshchekov et al., 2002; Nagel et al., 2003; Suzuki et al., 2003). The unique ability of channelrhodopsins to induce cell depolarization by blue light was fundamental for the emergence of optogenetics (Nagel et al., 2002, 2003).

In the following years, rhodopsin domains from channelrhodopsins and other photosensitive domains were increasingly employed to control key intracellular processes such as protein secretion, nuclear import, chromatin targeting, and gene expression (Kianianmomeni, 2014; Schmidt and Cho, 2015). Additionally, photoswitchable enzymerhodopsins have emerged as promising tools for optogenetics. In algae, this group includes histidine kinase rhodopsins (HKRs) (Kateriya et al., 2004; Luck et al., 2012) and rhodopsin guanylyl cyclases (RGCs) (Tian et al., 2018a).

With the increase in sequenced algal genomes and transcriptomes, many new light-sensitive proteins have been found. It is now clear that many algae possess multiple photoreceptor classes that work together to precisely perceive environmental stimuli. For instance, cryptochromes and rhodopsins may act in concert to regulate development. Further newly discovered photoreceptors are expanding the molecular toolbox for optogenetics and providing new insights into light-regulated cellular processes.

Previous reviews have covered different aspects of algal (Hegemann et al., 2001; Hegemann, 2008; Kianianmomeni and Hallmann, 2014; Duanmu et al., 2017; Shankar et al., 2022; Vierock and Hegemann, 2023) and plant photoreceptors (Lariguet and Dunand, 2005; Galvão and Fankhauser, 2015; Kong and Okajima, 2016; Parihar et al., 2016; Kowalik and Chen, 2017; Kottke et al., 2018; Pierik and Ballare, 2021), especially their chromophore (Galvão and Fankhauser, 2015; Ziegler et al., 2016; Kowalik and Chen, 2017; Tian, 2018) and domain structures (Hegemann, 2008; Kianianmomeni and Hallmann, 2015b; Kong and Okajima, 2016; Parihar et al., 2016; Kottke et al., 2018) and their applications in optogenetics (Dugué et al., 2012; Hegemann and Nagel, 2013; Hegemann and Sigrist, 2013; Kianianmomeni and Hallmann, 2014; 2015a; 2016).

## 5 The different types of algal photoreceptors

Light-sensitive proteins are widespread throughout the tree of life, including in animals, plants, algae, fungi, protists, archaea, and bacteria. These proteins enable organisms to sense environmental light and to modulate their growth, development, behavior, and reproduction accordingly. Photoreceptors play key roles in regulating adaptive light responses such as phototaxis and phototropism. They are also essential for various developmental processes, including the coordination of flowering time, sexual development, and adaptation to light-dark cycles. Additionally, the regulation of gene expression at different hierarchical levels by light-responsive photoreceptors provides a complex mechanism for controlling the abundance and functionality of the respective proteins. As a result, light exerts indirect control over a wide range of cellular and physiological processes.

**Figure 5:**
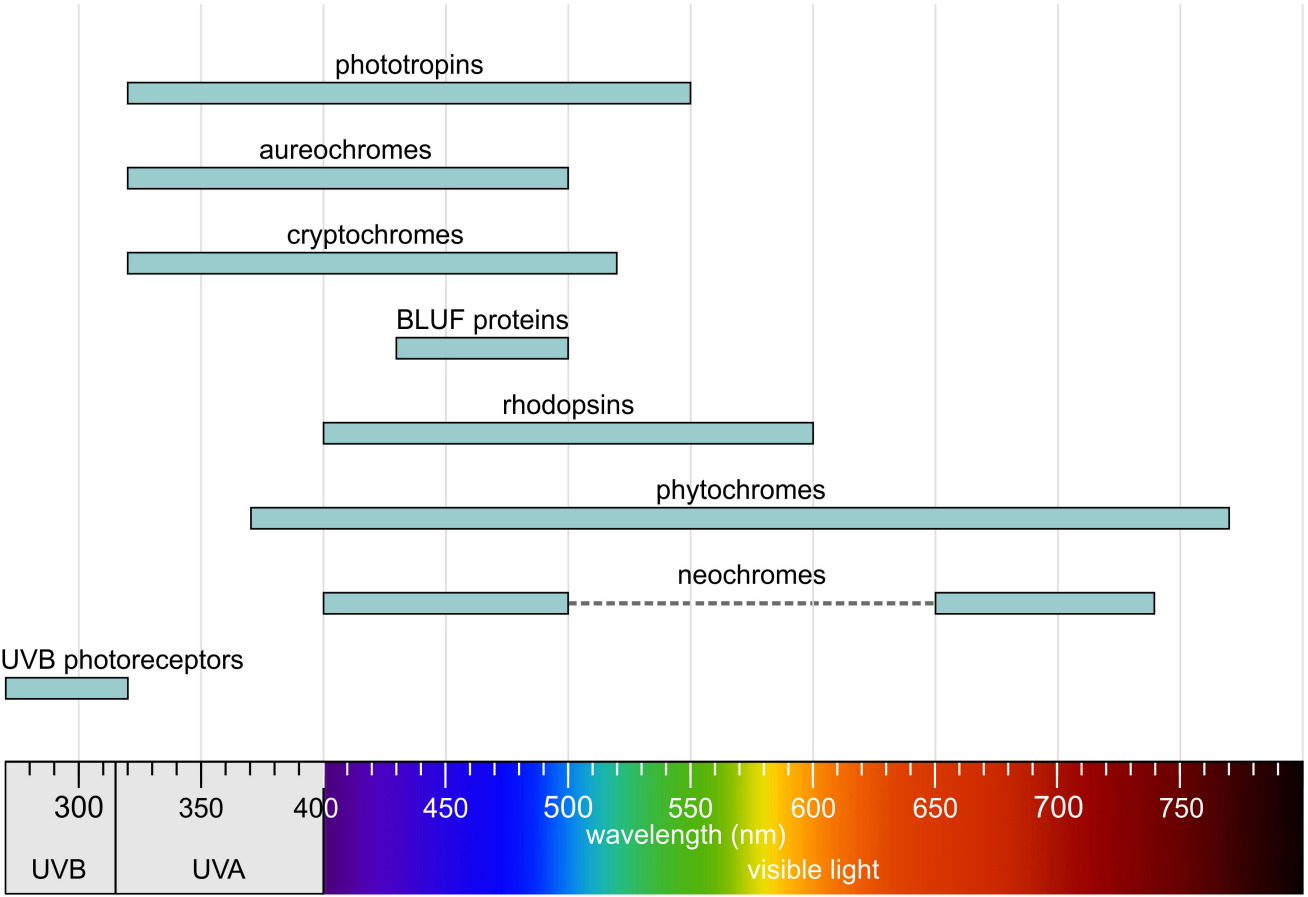
Absorption regions of photoreceptors. Redrawn and modified after Sager et al. (1988); Taiz and Zeiger (2002); Sullivan and Deng (2003); Lariguet and Dunand (2005); Rockwell et al. (2014); Galvão and Fankhauser (2015); Shankar et al. (2022), with additional information included.

All photoreceptors contain a chromophore—an organic molecule that absorbs light—bound to a protein (apoprotein) that determines specificity and mediates signal transduction. The primary function of photoreceptor proteins is to facilitate the absorption of photons and, upon absorption, to trigger a biological response either directly or indirectly. The protein component is responsible for converting light signals into biochemical reactions or signal transduction pathways; it also determines the specificity of the photoreceptor and influences the absorption wavelength of the chromophore. In most cases, photoreceptor proteins are capable of detecting a relatively broad range of absorption wavelengths found in sunlight. Overall, they cover the entire spectrum from UV-B to far-red light (**Figure 5**).

There are various ways to categorize photoreceptors (Kottke et al., 2018), for example by the wavelength of light absorbed, their mode of operation, the signaling pathways they serve, their evolutionary history, or the type of chromophore they use. In this context, the focus is on algal photoreceptors, which are classified into five main groups based on their chromophore. This classification also reflects structural similarities and evolutionary relationships. The five major groups are:

– Flavin-based receptors (phototropins, aureochromes, cryptochromes, BLUF proteins).
– Retinal-based receptors – rhodopsins (type-1 – microbial rhodopsins, type-2 – animal rhodopsins).
– Tetrapyrrole-based receptors (phytochromes).
– Hybrid receptors (neochromes).
– Proteins with UV absorption (UV-B photoreceptors).

It is noteworthy that only the retinal-based receptors are membrane-bound, while all others are soluble photoreceptors.

### 5.1 Flavin-based receptors

#### 5.1.1 Phototropins

Phototropins are flavin-based photoreceptors that are sensitive to blue light (**Figure 5**) and are widespread in both algae and plants (Losi and Gärtner, 2012; Suetsugu and Wada, 2013; Christie et al., 2015; Li et al., 2015b; Okajima, 2016; Hart and Gardner, 2021; Huq et al., 2024). They were first discovered in *Arabidopsis thaliana* in connection with blue light-dependent phosphorylation (Huala et al., 1997; Christie and Briggs, 2001; Christie, 2007). The basic mechanism of signal transduction is conserved in both algae and plants, even though the sequence identity is only 30–40% (Onodera et al., 2005; Kianianmomeni and Hallmann, 2014). Phototropins are involved in regulating various light-dependent processes such as phototaxis, chloroplast movement, and photomorphogenesis. In algae, phototropins are associated with evolutionary adaptation to light-dependent aquatic environmental conditions (Kami et al., 2010). Phototropins also influence the expression of genes encoding enzymes for chlorophyll and carotenoid biosynthesis in response to blue light (Im et al., 2006).

Only a single phototropin, PHOT, has been identified in the green algae *Volvox carteri* (**Figure 3**) and *Chlamydomonas reinhardtii* (**Figure 3**) (Huang et al., 2002; Prochnik et al., 2010). This single phototropin is involved in the regulation of photoprotection (Petroutsos et al., 2016), eyespot formation (Trippens et al., 2012), reproduction (Huang and Beck, 2003), and starch accumulation (Yuan et al., 2025). In *Volvox*, phototropin accumulation is higher in the small somatic cells than in the large reproductive cells, suggesting a link between PHOT expression and the mechanism controlling cell size during *Volvox* development (Kianianmomeni and Hallmann, 2014). Due to gene duplication, higher plants possess two phototropin isoforms, PHOT1 and PHOT2 (Li et al., 2015b; Hart and Gardner, 2021).

**Figure 6:**
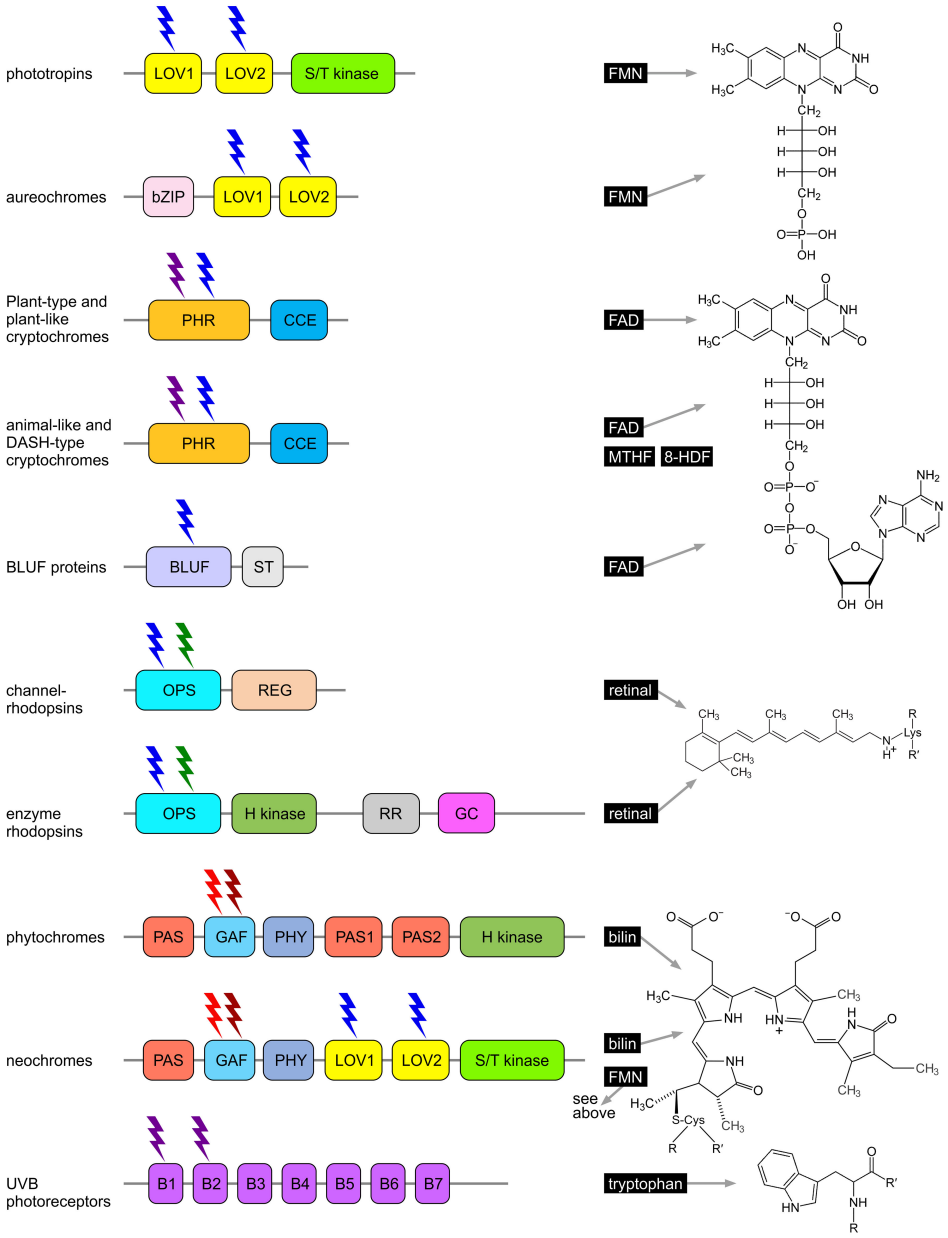
Algal photoreceptors and their chromophores. The domain structure of each photoreceptor protein is shown on the left, and the chemical structure of the associated chromophore is depicted on the right. Phototropins, aureochromes, channelrhodopsins, and cryptochromes primarily absorb blue light, while UVB receptors (UVR8) are sensitive to UVB light. Neochromes and animal-like cryptochromes respond to both red and blue light; enzyme rhodopsins are receptive to both blue and green light; and phytochromes respond to red and far-red light, with some also sensitive to orange, blue, and green light. 8-HDF, 8-hydroxy-7,8-didemethyl-5-deazariboflavin; B1-7, β-propeller domains; bilin, phycocyanobilin (PCB); BLUF, blue light using FAD; bZIP, basic region/leucine zipper; CCE, cryptochrome C-terminal extension (CCT, CTE); FAD, flavin adenine dinucleotide; FMN, flavin mononucleotide; GAF, cGMP phosphodiesterase/adenyl cyclase Fh1A; GC, guanylyl cyclase; H kinase, histidine kinase; LOV, light, oxygen, voltage; MTHF, 5,10-methenyltetrahydrofolate; OPS, opsin; PAS, PER-ARNT-SIM; PHR, photolyase homology region; PHY, phytochrome-specific; REG, regulator; retinal, all-trans-retinal; RR, response regulator receiver; ST, signal transduction; S/T kinase, serine/threonine kinase. Redrawn, modified, and supplemented from Galvão and Fankhauser (2015); Kong and Okajima (2016); Parihar et al. (2016); Shankar et al. (2022).

Phototropins have a modular structure; in algae, they consist of two N-terminal light, oxygen, or voltage (LOV) domains and a C-terminal serine/threonine kinase (S/T kinase) domain (**Figure 6**) (Crosson and Moffat, 2001; Huang and Beck, 2003; Kottke et al., 2006; Aihara et al., 2008; Losi and Gärtner, 2012; Christie et al., 2015). The LOV domains are responsible for blue light perception, with each binding a flavin mononucleotide (FMN) as a chromophore (**Figure 6**) (Crosson et al., 2003; Kottke et al., 2003; Kennis et al., 2004; Losi et al., 2004; Briggs et al., 2007; Losi, 2007; Hoffman et al., 2018; Losi et al., 2018; Flores-Ibarra et al., 2024; He et al., 2025a).

Upon absorption of blue light, a covalent bond forms between the FMN and a cysteine residue, triggering a conformational change in the protein. In particular, LOV1 plays a supportive role in stabilizing the protein and regulating its interactions with other molecules, while the conformational change in LOV2 upon light activation enables activation of the kinase domain. The S/T kinase domain catalyzes autophosphorylation and subsequently transmits the signal by phosphorylating target proteins at serine and threonine residues. This domain is crucial for propagating the light signal by activating downstream signaling cascades (Christie et al., 1998, 2002; Hart and Gardner, 2021; Flores-Ibarra et al., 2024).

The 3D structure of phototropins has only been partially elucidated, as the structure of the light-dependent S/T kinase domain has not yet been fully resolved. However, the structures of the LOV domains from phototropins in both plants and algae—under dark and light conditions—have been determined using crystallographic techniques (Christie et al., 1999; Crosson and Moffat, 2002; Fedorov et al., 2003; Halavaty and Moffat, 2007; Flores-Ibarra et al., 2024; Gotthard et al., 2024). The LOV1 and LOV2 domains belong to the same family as PER-ARNT-SIM (PAS) domains. LOV domains are composed of five antiparallel β-strands flanked by two α-helices, creating a specific binding pocket for the flavin chromophore (Crosson and Moffat, 2001; 2002;Nash et al., 2011; Zoltowski et al., 2013). The conformational change that occurs after formation of the light-induced covalent bond between the cysteine residue of the LOV domain and the FMN particularly affects the so-called Jα helix, which is associated with the LOV2 domain (Crosson et al., 2003; Harper et al., 2003, 2004; Halavaty and Moffat, 2007; Vaidya et al., 2011). The Jα helix becomes accessible, facilitating transmission of the signal to the S/T kinase domain. The S/T kinase domain belongs to the family of serine/threonine protein kinases and exhibits the typical bilobal structure of these enzymes. The catalytic core consists of a small N-terminal lobe, which typically features a β-sheet and an α-helix, and a larger C-terminal lobe that is predominantly α-helical. Activation of this core is triggered by light-induced activation of the LOV2 domain (Matsuoka and Tokutomi, 2005; Jones and Christie, 2008; Hart and Gardner, 2021).

#### 5.1.2 Aureochromes

Aureochromes are flavin-based photoreceptors that are sensitive to blue light (**Figure 5**) (Suetsugu and Wada, 2013; Takahashi, 2016; Essen et al., 2017; Kroth et al., 2017; Matiiv and Chekunova, 2018; Coesel, 2024; Im et al., 2024). More specifically, they are light-controlled transcription factors that regulate genes important for algal adaptation to light, such as those involved in cell movement (phototaxis), chloroplast arrangement, or light-dependent differentiation. Aureochromes also contribute to optimizing photosynthesis under varying light conditions (Schellenberger Costa et al., 2013; Kroth et al., 2017; Matiiv and Chekunova, 2018).

Aureochromes possess a modular structure, combining sensor and regulatory functions within a single molecule (Mitra et al., 2012; Banerjee et al., 2016a; Heintz and Schlichting, 2016; Hepp et al., 2020). They consist of C-terminal light, oxygen, or voltage (LOV) domains and an N-terminal basic leucine zipper (bZIP) domain (**Figure 6**). The arrangement of the sensor and regulatory domains is therefore reversed compared to many other photoreceptors (Herman et al., 2013; Banerjee et al., 2016b; Kalvaitis et al., 2019; Coesel, 2024). The LOV domains are responsible for blue light perception, each binding a flavin mononucleotide (FMN) as a chromophore (Takahashi et al., 2007; Herman et al., 2013; Coesel, 2024). As in phototropins, LOV domains are composed of five antiparallel β-strands flanked by two α-helices (Mitra et al., 2012; Banerjee et al., 2016a; Heintz and Schlichting, 2016; Hepp et al., 2020), forming a specific binding pocket for the flavin chromophore. Upon absorption of blue light and formation of a light-induced covalent bond between a specific cysteine residue in the LOV domain and the FMN, a conformational change occurs, particularly affecting the Jα helix associated with the LOV domain. This activates the bZIP domain, which then binds DNA in a sequence-specific manner and functions as a transcription factor. The activated bZIP domain thus regulates the expression of target genes in a light-dependent manner (Mitra et al., 2012; Banerjee et al., 2016a; Heintz and Schlichting, 2016; Hepp et al., 2020).

Aureochromes were first identified in the alga *Vaucheria frigida* (Xanthophyta, **Figure 4**) (Takahashi et al., 2007; Matiiv and Chekunova, 2018), where they play an important role in the blue light-dependent regulation of sexual organ development and the induction of branching (Takahashi et al., 2007). Subsequently, aureochromes were also found in the diatoms (**Figure 4**) *Phaeodactylum tricornutum* (**Figure 3**), *Thalassiosira pseudonana* (**Figure 3**), and *Thalassiosira oceanica*; the eustigmatophyte (**Figure 4**) *Nannochloropsis gaditana* (**Figure 3**); the raphidophyte *Chattonella antiqua*; the golden-brown alga *Ochromonas danica*; and the brown algae (**Figure 4**) *Ectocarpus siliculosus* (**Figure 3**) and *Fucus distichus*, among others (Armbrust et al., 2004; Bowler et al., 2008; Ishikawa et al., 2009; Cock et al., 2010; Rayko et al., 2010; Lommer et al., 2012; Radakovits et al., 2012). A common feature of these organisms is that yellow-green algae (Xanthophyta), diatoms (Bacillariophyta), eustigmatophytes (Eustigmatophyta), raphidophytes (Raphidophyta), golden-brown algae (Chrysophyta), and brown algae (Phaeophyta) all belong to the stramenopiles. To date, aureochromes appear to be restricted to photosynthetic stramenopiles (ochrophytes) (Ishikawa et al., 2009; Takahashi, 2016; Kroth et al., 2017; Coesel, 2024; Im et al., 2024) and have not been detected in nonphotosynthetic stramenopile lineages such as oomycetes (e.g., *Phytophthora*), opalinids (e.g., *Opalina*), or bigyra (e.g., *Blastocystis*), nor in any other eukaryotic groups. This distribution suggests that aureochromes either evolved in the common ancestor of ochrophytes after secondary endosymbiosis with a red alga, or were lost early in nonphotosynthetic stramenopiles. The strict correlation with photosynthetic lineages is consistent with their role in regulating light-dependent morphogenetic and photosynthetic processes (Takahashi et al., 2007; Takahashi, 2016; Kroth et al., 2017; Madhuri et al., 2024; Zhang et al., 2024a).

#### 5.1.3 Cryptochromes

Cryptochromes are flavin-based photoreceptors that primarily absorb in the UV-A and blue light ranges. They covalently bind flavin adenine dinucleotide (FAD) as a catalytic chromophore (Lin and Todo, 2005; Yu et al., 2010; Chaves et al., 2011; Losi and Gärtner, 2012; Essen et al., 2017; Kottke et al., 2017; Petersen et al., 2021; Deoliveira and Crane, 2024). However, depending on the redox state of the flavin, they can also absorb red light (Beel et al., 2012; Oldemeyer et al., 2019; Kiontke et al., 2020). Some cryptochromes are also capable of binding 5,10-methenyltetrahydrofolate (MTHF) or 8-hydroxy-7,8-didemethyl-5-deazariboflavin (8-HDF) as a second chromophore and antenna pigment (Sancar, 2003; Chaves et al., 2011). Cryptochromes, together with photolyases, form the cryptochrome/photolyase family (CPF), which includes cryptochrome photoreceptors as well as cyclobutane pyrimidine dimer (CPD) photolyases and 6–4 photolyases—light-dependent enzymes involved in the repair of UV-damaged DNA (Coesel et al., 2009; Heijde et al., 2010; Losi and Gärtner, 2012; Juhas et al., 2014; Franz et al., 2018; Yan et al., 2024). However, most cryptochromes lack DNA repair activity. Nevertheless, all cryptochromes possess a conserved photolyase homology region (PHR) and a C-terminal extension of varying length, known as the cry C-terminal extension (CCE) domain (**Figure 6**). The PHR domain binds the chromophore (Lin and Todo, 2005; Fraikin et al., 2023). Light absorption triggers a redox reaction in the chromophore (e.g., FAD), and the resulting conformational changes are transmitted to the CCE domain, which acts as the effector domain for signal transduction. The CCE domain interacts with specific target proteins and mediates cellular responses characteristic of the particular cryptochrome (Yu et al., 2010; Kondoh et al., 2011; Liu et al., 2016; Petersen et al., 2021). Cryptochromes are broadly categorized into plant-type, plant-like, animal-like, and DASH cryptochromes (Kottke et al., 2017; Rredhi et al., 2021; Shankar et al., 2022). The type and number of cryptochromes vary greatly among different algal species (Fortunato et al., 2015; Kottke et al., 2017).

Plant-type and plant-like cryptochromes, as the name suggests, closely resemble cryptochromes found in higher plants (Müller et al., 2017; Wang and Lin, 2025). Plant-type cryptochromes are primarily responsible for regulating growth processes and synchronizing circadian rhythms. They are also involved in the regulation of phototaxis and influence the expression of light-regulated genes. In *Chlamydomonas*, the blue light-regulated, plant-type cryptochrome pCRY (formerly designated CPH1) has been shown to regulate key aspects of the circadian clock and life cycle progression (Müller et al., 2017). Also noteworthy is a dual photoreceptor known as dualchrome, a chimeric protein that contains both a plant-type cryptochrome and a phytochrome domain (Makita et al., 2021). This orange/far-red and blue light photoreceptor originates from the marine picoplankton alga *Pycnococcus provasolii*.

Plant-like cryptochromes also show high similarity to plant cryptochromes but are distinguished by species-specific sequence characteristics. Plant-like cryptochromes are involved in entraining the circadian clock (Galvão and Fankhauser, 2015), synchronizing biological rhythms with day length and light availability to regulate processes such as cell division, photosynthesis, and growth. They may act directly or indirectly on the expression of genes associated with light-dependent processes. By upregulating or downregulating genes related to light utilization and protective mechanisms, such as during light stress, plant-like cryptochromes enable algae to adapt to changing light conditions. Moreover, plant-like cryptochromes can interact with other photoreceptors, such as phototropins and phytochromes, to allow responses to a broader spectrum of light (**Figure 5**) (Lin, 2002; Wang et al., 2014; D'amico-Damião and Carvalho, 2018; Wang and Lin, 2025).

Animal-like cryptochromes exhibit the highest sequence similarity—and thus the closest evolutionary relationship—to cryptochromes from animals (Zou et al., 2017; Mat et al., 2024). Animal-like cryptochromes are responsive to nearly the entire visible spectrum, including red light (**Figure 5**). These cryptochromes may activate signaling pathways that are particularly specific for the perception of light quality and light dynamics, whereas plant-like cryptochromes are more specialized for synchronization with the daylight cycle. Animal-like cryptochromes play a key role in the sexual life cycle and, in combination with plant cryptochromes, can serve as negative regulators of mating ability, in contrast to the function of phototropin (Zou et al., 2015). However, animal-like cryptochromes can positively regulate vegetative germination in algae. In *Chlamydomonas*, the animal-like cryptochrome aCRY modulates the light-dependent expression of various genes encoding proteins involved in chlorophyll and carotenoid biosynthesis, light-harvesting complexes, nitrogen metabolism, cell cycle control, and the circadian clock (Beel et al., 2012).

DASH-type cryptochromes (CRY-DASHs) are evolutionarily most closely related to 6–4 photolyases and animal cryptochromes, but their biological function remains uncertain. They have a maximal absorption peak in the UV-A range and use 5,10-methenyltetrahydrofolate (MTHF) as an antenna chromophore. In addition to their role as photoreceptors, they have been shown to repair photodamaged single-stranded and loop-structured double-stranded DNA *in vitro* (Selby and Sancar, 2006; Pokorny et al., 2008; Beel et al., 2012). As UV photoreceptors, they are also believed to serve an important function: DASH cryptochromes may act as UV-A sensors required to balance components of the photosynthetic machinery and support photoautotrophic growth (Rredhi et al., 2021). Since UV-A is not detected by the photochemical pigments of photosystem I and II, a central UV-A receptor in the chloroplast may be necessary to regulate the photosynthetic machinery independently of the chloroplast’s pigments, which mainly absorb in the blue and red regions of the visible spectrum (Rredhi et al., 2021).

#### 5.1.4 BLUF proteins

BLUF (blue light using FAD) proteins are photoreceptors that primarily absorb light in the blue spectral range, typically at wavelengths around 430–500 nm (**Figure 5**). They non-covalently bind flavin adenine dinucleotide (FAD) as a catalytic chromophore (Gomelsky and Klug, 2002; Losi and Gärtner, 2012; Park and Tame, 2017; Losi et al., 2018; He et al., 2025a). BLUF proteins are found in certain algae, many bacteria, and some fungi. The blue light detected by BLUF proteins serves as a signal to control processes such as phototaxis, photomorphogenesis, and the regulation of cellular activity in response to light. These proteins may also be involved in regulating the expression of genes responsible for adaptation to light conditions, including genes for photosynthesis and protection against light stress (Park and Tame, 2017; Kaushik et al., 2019). A BLUF domain was first identified in the prokaryote *Rhodobacter* sp*haeroides* (Rhodobacterales), which belongs to the purple non-sulfur photosynthetic bacteria, where this domain regulates the expression of photosynthesis genes (Gomelsky and Kaplan, 1998; Gomelsky and Klug, 2002). In *Euglena* (Euglenozoa) (**Figure 3**, **Figure 4**), BLUF proteins are known to be involved in photophobic responses (Iseki et al., 2002). The central light-sensitive unit of BLUF proteins is the N-terminal BLUF domain, which contains FAD as the chromophore (**Figure 6**). The domain consists of a conserved ferredoxin-like β-α-β-β-α-β fold (Jung et al., 2005, 2006; Wu and Gardner, 2009; Kennis and Mathes, 2013), which is important for signal transduction (Fujisawa and Masuda, 2018). Absorption of blue light induces a conformational change in the BLUF domain (Mathes and Götze, 2015), characterized by a typical and reversible 10 nm red shift in the absorption spectrum, while the chromophore remains in the oxidized state. A C-terminal signal transduction (ST) domain serves as an effector domain for this conformational change and can function as an enzyme domain. The ST domain is responsible for interactions with other proteins (Mathes and Götze, 2015; Kaushik et al., 2019; Taguchi et al., 2024).

### 5.2 Retinal-based receptors

#### 5.2.1 Rhodopsins

Rhodopsins, also known as retinylidene proteins, are retinal-based, membrane-bound photoreceptors that can be sensitive to wavelengths ranging from violet to orange light (**Figure 5**) (Nathans, 1992; Zhang et al., 2011; Nagata and Inoue, 2021; Rozenberg et al., 2021; De Grip and Ganapathy, 2022). However, the majority of algal rhodopsins absorb light in the 450–550 nm range (blue to green light) (Hegemann, 2008; Kato et al., 2015; Mcisaac et al., 2015). The absorption spectra of different rhodopsins vary due to subtle differences in the structure of the retinal chromophore or the surrounding protein environment. Rhodopsins are found in all three domains of life: bacteria, archaea, and eukarya. They are involved in a range of essential light-dependent processes, including phototaxis, circadian rhythms, regulation of the cell cycle, and stress responses. Rhodopsins can function as monomers, but they frequently also form dimers or oligomers. Retinal forms a covalent linkage via a protonated Schiff base with a conserved lysine located in transmembrane helix 7 (TM7). Based on their retinal configuration, rhodopsins are divided into two major groups: type 1 (microbial rhodopsins) and type 2 (animal rhodopsins). In type 1 rhodopsins, the protein environment is evolutionarily optimized for light-induced retinal isomerization from all-trans to 13-cis. In contrast, type 2 rhodopsins are optimized for isomerization from 11-cis to all-trans (Nakanishi, 1991; Spudich et al., 2000). The photon-absorbing retinal chromophore, which is the aldehyde form of vitamin A, is derived from cleaved β-carotene and is covalently linked to the opsin apoprotein (Spudich et al., 2000). When light strikes the retinal, photoisomerization occurs. This conformational change stores the energy of the photon and transfers it as mechanical energy to the opsin apoprotein, initiating downstream reactions. All rhodopsins share a common architecture of seven transmembrane α-helices, with the N-termini oriented outward and the C-termini facing inward (Soppa, 1994; Spudich et al., 2000; Pierce et al., 2002). The retinal chromophore is attached via a Schiff base linkage to the ϵ-amino group of a lysine residue located in the middle of the seventh transmembrane helix. Typically, the retinal Schiff base is protonated and exists in the 15-anti configuration. Changes in the protonation state are essential for the signaling or transport activities of rhodopsins. Despite these structural similarities, it is notable that type 1 and type 2 rhodopsins show no recognizable sequence similarity, as they are the result of convergent evolution (Soppa, 1994; Kojima and Sudo, 2023).

Type-2 rhodopsins (animal-type) are mentioned here only for the sake of completeness. They are characteristic of animals and have not been detected in any algal lineage, including dinoflagellates. These rhodopsins, which have been known the longest, belong to the superfamily of G-protein-coupled receptors (GPCRs) and were first identified as the visual pigment in the rod photoreceptor cells of vertebrate eyes (Spudich et al., 2000; Pierce et al., 2002; Palczewski, 2006; Rosenbaum et al., 2009; Mat et al., 2024). Unlike most GPCRs, which are activated by the binding of small ligands, rhodopsins are activated by the light-induced isomerization of their chromophore, which in turn initiates the G-protein-mediated signaling cascade. The membrane-bound rhodopsin thus serves as a sensor that activates transmembrane or soluble transducers. In addition to their role in visual phototransduction, type-2 rhodopsins are also involved in non-visual phototransduction, regulation of the circadian clock, and as enzymes that catalyze the isomerization of photopigments (photoisomerases) (Shichida and Matsuyama, 2009; Schmidt et al., 2011).

**Figure 7:**
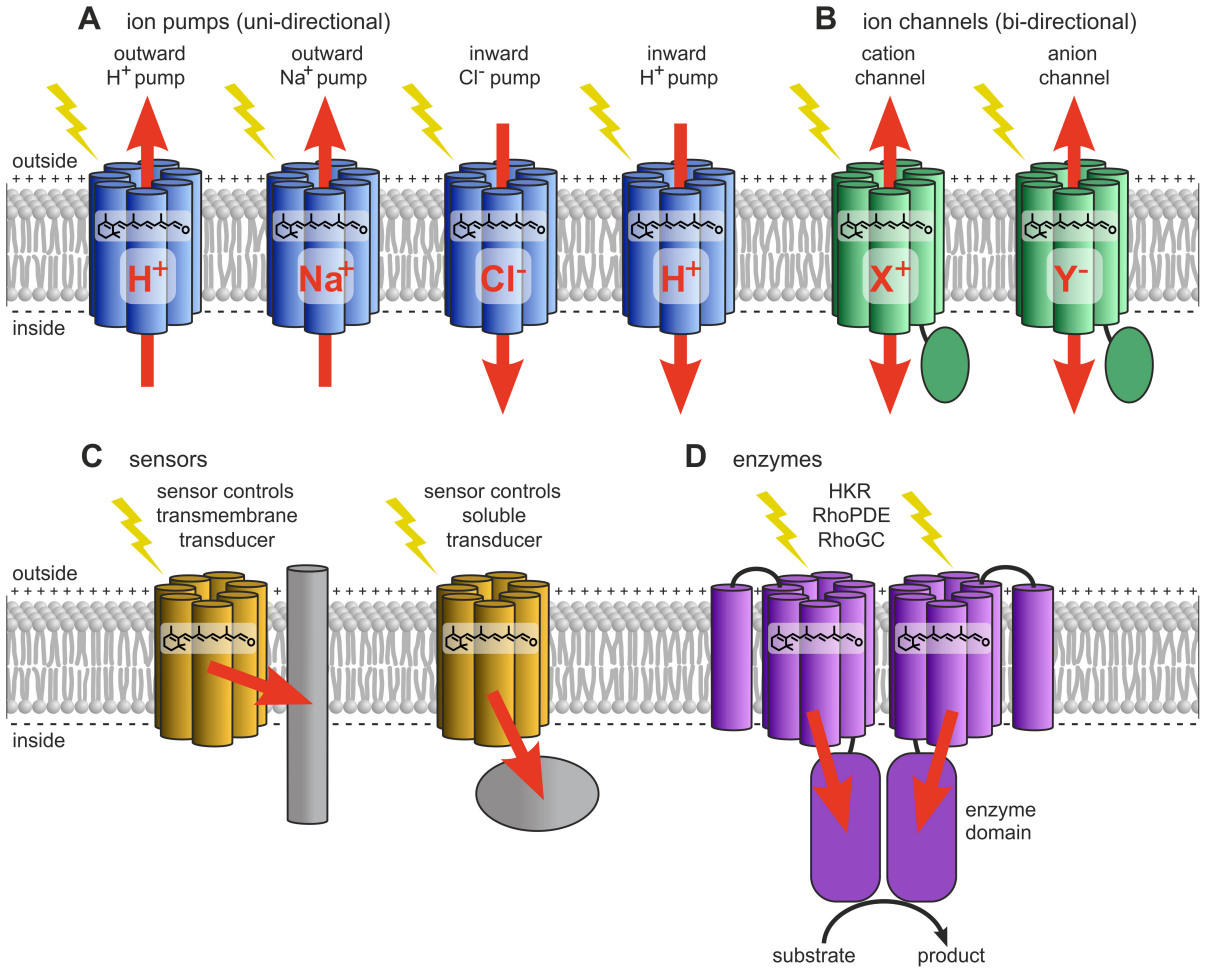
Type-1 rhodopsins (microbial type). **(A)** Ion-pumping rhodopsins include outward and inward-directed proton pumps, outward sodium ion pumps, and inward chloride ion pumps. **(B)** Ion-channeling rhodopsins comprise cation channelrhodopsins (CCRs) and anion channelrhodopsins (ACRs). **(C)** Sensory rhodopsins regulate transmembrane or soluble cytosolic transducers. **(D)** Enzyme rhodopsins include histidine kinase rhodopsins (HKRs), rhodopsin phosphodiesterases (RhoPDEs), and rhodopsin guanylyl cyclases (RhoGCs). Redrawn and modified after Zhang et al. (2011); Rozenberg et al. (2021); Tsunoda et al. (2021).

Type-1 rhodopsins (microbial-type) (**Figure 7**) are found in algae as well as in bacteria, fungi, archaea, protists (such as choanoflagellates), and even viruses (Ruiz-González and Marín, 2004; Grote et al., 2014; Govorunova et al., 2017; Zabelskii et al., 2020; Nagata and Inoue, 2021; Rozenberg et al., 2021; De Grip and Ganapathy, 2022; Govorunova et al., 2022; Govorunova and Sineshchekov, 2023; Vlasova et al., 2024). Because they were originally discovered in archaea (*Halobacterium*), they were formerly referred to as archaeal rhodopsins (Oesterhelt and Stoeckenius, 1971).

In algae, type-1 rhodopsins are widespread, and each algal species appears to possess a whole series of these rhodopsins. For example, at least seven rhodopsin-based photoreceptors have been identified in each of the green algae *Chlamydomonas reinhardtii* and *Volvox carteri* (Hegemann, 2008; Kianianmomeni and Hallmann, 2014; 2015b). Dinoflagellates likewise encode multiple type-1 rhodopsins (often termed proteorhodopsins or rhodopsin-like proteins), which are present in both photosynthetic (e.g., *Prorocentrum*) and heterotrophic (e.g., *Oxyrrhis*) species and are thought to function in light-driven proton pumping, sensory roles, and possibly energy supplementation (Ruiz-González and Marín, 2004; Slamovits et al., 2011; Shi et al., 2015).

Based on their biological function, type-1 rhodopsins can be classified as light-dependent outward and inward ion pumps, ion channels, sensory rhodopsins, and enzyme rhodopsins (**Figure 7**) (Klare et al., 2008; Ernst et al., 2014; Rozenberg et al., 2021).

Light-driven ion pumps transport ions in a single direction (**Figure 7**). They are subdivided into outward and inward-directed proton pumps, outward sodium ion pumps, and inward chloride ion pumps (Kandori, 2015).

Light-driven outward-directed proton pumps (**Figure 7**), a type of cation pump, are typically involved in energy generation (Oesterhelt and Stoeckenius, 1973; Inoue et al., 2016). In proton pumps such as bacteriorhodopsin (from *Halobacterium*) and proteorhodopsin (from marine bacteria), light energy is used to pump protons out of the cell, thereby creating an electrochemical proton gradient across the membrane. This gradient is then utilized by ATP synthase to produce ATP as the protons flow back into the cell.

Ion-transporting rhodopsins also include the less common inward-directed proton pumps. Schizorhodopsins (SzRs) are examples of such light-driven inward proton pumps (Inoue et al., 2020; Brown, 2022). They have been found in *Schizochytrium* (Thraustochytriaceae) and other members of the Thraustochytriaceae, which are unicellular heterotrophic marine protists of the Stramenopile group, often considered non-photosynthetic microalgae. These inward proton pumps are also present in Asgard archaea, a superphylum that appears to include the closest archaeal relatives of eukaryotes.

Additionally, there are rare xenorhodopsins (XeRs), which transport protons inward while simultaneously transporting anionic substrates (e.g., Cl^-^, NO_3_^-^, or other negative ions) outward, i.e., in the opposite direction (Shevchenko et al., 2017; Inoue et al., 2018; Weissbecker et al., 2021; Brown, 2022).

Light-driven outward-directed sodium ion pumps (**Figure 7**) (Inoue et al., 2013), another type of cation pump, generate a sodium gradient across the membrane. The resulting electrochemical gradient can be utilized for various physiological processes, such as ATP synthesis or osmotic adaptation under extreme environmental conditions. The characterization of the light-driven sodium pump from *Krokinobacter eikastus*, rhodopsin 2 (KR2), demonstrated for the first time that light-driven cation transport is not limited to protons (Inoue et al., 2013).

Light-driven inward-directed chloride ion pumps (**Figure 7**) (Mukohata and Kaji, 1981), a type of anion pump, are found, for example, in the halorhodopsins of halophilic archaea and bacteria. These pumps actively transport chloride ions across cell membranes, contributing to the maintenance of osmotic balance and also playing a role in energy production.

Light-regulated ion channels (**Figure 7**) enable cells to adapt to changing light conditions (Spalding, 2000). Typically, ion channels allow ions to passively flow down their concentration gradient across the membrane, meaning that ions can move in both directions. This group includes cation channelrhodopsins (CCRs), bacteriorhodopsin-like cation channelrhodopsins, and anion channelrhodopsins (ACRs).

Light-gated cation channelrhodopsins (CCRs) (**Figure 7**, **Figure 6**) (Nagel et al., 2002, 2003) selectively permit the passage of H^+^, Na^+^, K^+^, and Ca^2+^ ions in response to light. Illumination, particularly in the blue spectrum, induces a conformational change in the retinal cofactor within the protein. This change opens the channel, allowing cations to flow bidirectionally along their electrochemical gradient. In the chlorophyte *Chlamydomonas*, two plasma membrane-localized channelrhodopsins, ChR1 and ChR2, regulate positive and negative phototaxis as well as photophobic responses (Ridge, 2002; Sineshchekov et al., 2002; Berthold et al., 2008). ChR1 and ChR2 were the first channelrhodopsins to be discovered (Nagel et al., 2002, 2003). ChR1 has an absorption maximum at 480 nm (blue) and is selective for protons, whereas ChR2 has a similar absorption maximum at 470 nm (blue) but displays broader specificity, being conductive for monovalent and divalent cations such as Na^+^, K^+^, and Ca^2+^. These channelrhodopsins are localized in the plasma membrane above the eyespot (Foster and Smyth, 1980; Foster et al., 1984). Light-induced cation influx causes depolarization of the plasma membrane at the eyespot (Berthold et al., 2008). This depolarization is then transmitted to the peri-flagellar plasma membrane, where it activates voltage-gated calcium channels (VGCCs), resulting in an immediate calcium influx and an increase in intracellular Ca^2+^ concentration (Berthold et al., 2008). The local rise in Ca^2+^ ions in the peri-flagellar membrane region is crucial for regulating flagellar movement by modulating the beating frequency and angle of the flagella (Kamiya and Witman, 1984). This process enables directed movement of the alga toward a light source (positive phototaxis) or away from it (negative phototaxis) (Rüffer and Nultsch, 1998; Isogai et al., 2000; Wakabayashi et al., 2021). Photophobic responses (light avoidance behaviors) are triggered by sudden strong illumination or abrupt changes in light intensity, where a pronounced Ca^2+^ influx leads to a rapid alteration in flagellar activity, allowing the alga to quickly correct its movement (Hyams and Borisy, 1978; Bessen et al., 1980; Fujiu et al., 2009). The influx of cations, particularly protons, also lowers intracellular pH, thereby regulating enzymatic activities and other cellular responses.

**Figure 8:**
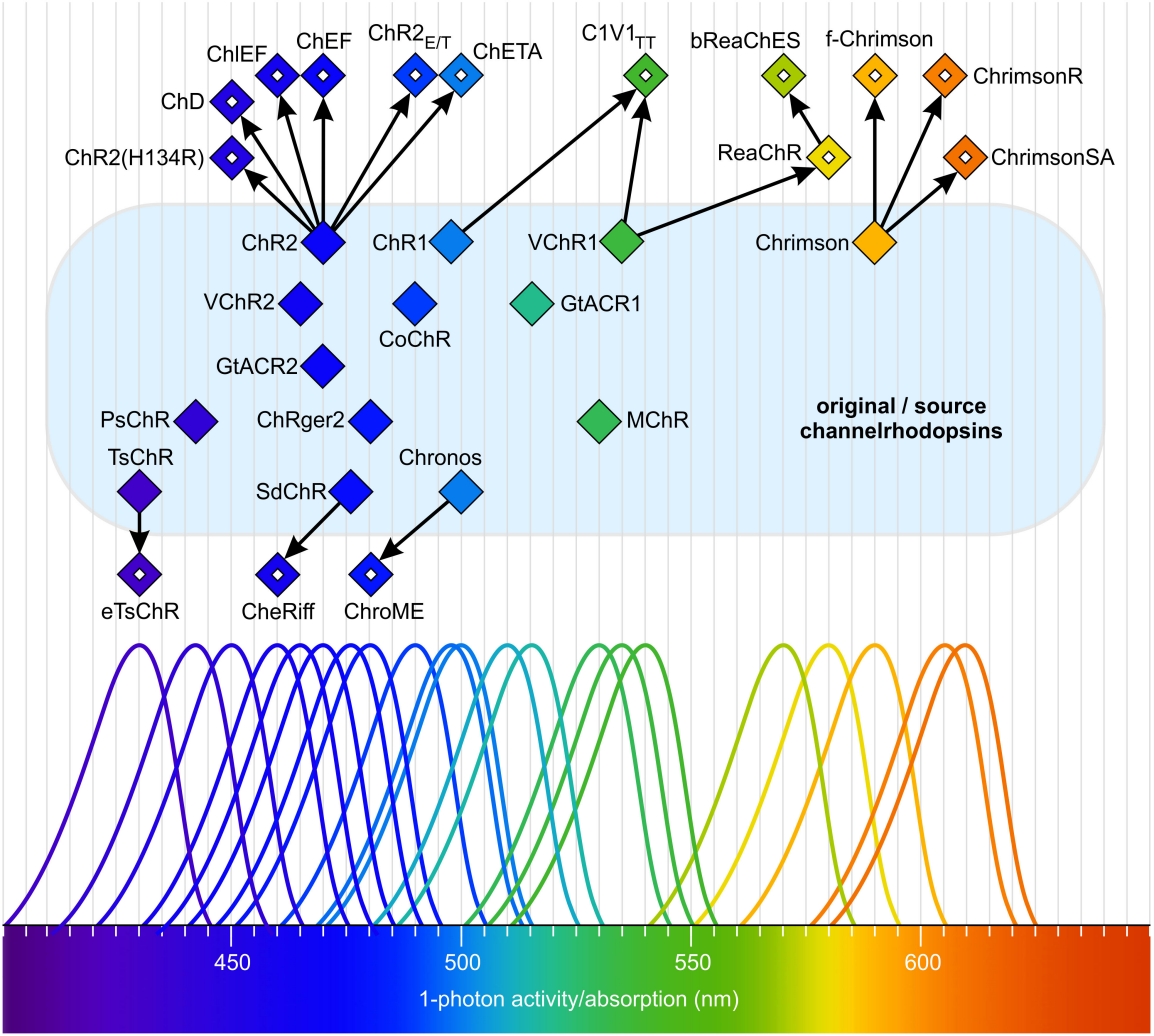
Natural and mutated channelrhodopsins span a wide range of the visible spectrum. Filled diamonds indicate the absorption maxima and single-photon activity of natural channelrhodopsins. Open diamonds represent the absorption maxima and single-photon activity of mutant channelrhodopsins derived from the original forms. All natural (original/source) channelrhodopsins are located on a light blue surface. Arrows indicate the relationships between origins. The schematic absorption spectra are intended to highlight the maxima and to illustrate the approximate portion of the total spectrum covered. Redrawn, modified, and supplemented after Mattis et al. (2011); Chuong et al. (2014); Klapoetke et al. (2014); Venkatachalam and Cohen (2014); Kim et al. (2015); Olofsson et al. (2015); Wietek and Prigge (2016); Rindner and Lur (2023); Vierock and Hegemann (2023).

Relatives of ChR1 and ChR2 from *Chlamydomonas reinhardtii* have been identified in several other chlorophytes (**Figure 8**), including VChR1 and VChR2 in *Volvox carteri*, Chrimson (also known as CnChR) in *C. noctigama*, Chronos (ShChR) in *Stigeoclonium helveticum*, CoChR in *Chloromonas oogama*, TsChR in *Tetraselmis striata*, TcChR in *T. cordiformis*, SdChR in *Scherffelia dubia*, MChR in *Mesostigma viride*, CsChR in *Chloromonas subdivisa*, AgChR in *Asteromonas gracillis*-B, NsChR in *Neochlorosarcina* sp., BsChR in *Brachiomonas submarina*, CbChR in *C. bilatus*-A, PsChR in *Proteomonas sulcata*, PsChR in *Platymonas subcordiformis*, HpChR1 in *Haematococcus pluvialis*, HdChR in *H. droebakensis*, CaChR1 in *C. augustae*, CyChR1 in *C. yellowstonensis*, and CraChR2 in *C. raudensis* (Kianianmomeni et al., 2009; Govorunova et al., 2011; Mattis et al., 2011; Govorunova et al., 2013; Chuong et al., 2014; Klapoetke et al., 2014; Venkatachalam and Cohen, 2014; Kim et al., 2015; Olofsson et al., 2015; Wietek and Prigge, 2016; Bi et al., 2022; Rindner and Lur, 2023; Vierock and Hegemann, 2023). There are a few reports in algae on cation channelrhodopsins with naturally increased selectivity for specific ions that would be of interest for optogenetic applications. One example is the enhanced sodium conductance of PsChR in *Tetraselmis subcordiformis* (Sineshchekov et al., 2013; Coli et al., 2024).

Cation channelrhodopsins from cryptophyte algae (Cryptophyta, **Figure 4**) differ from the light-gated cation channelrhodopsins described in chlorophytes (Govorunova et al., 2016; Shigemura et al., 2019). These unique channelrhodopsins are more homologous to haloarchaeal rhodopsins, such as the proton-pumping bacteriorhodopsin, than to chlorophyte channelrhodopsins (Govorunova et al., 2016; Shigemura et al., 2019). Furthermore, chlorophyte and cryptophyte channelrhodopsins exhibit different structural features, indicating that cation channelrhodopsins have evolved independently through convergent evolution.

Light-gated anion channelrhodopsins (ACRs) (**Figure 7**) (Govorunova et al., 2015; Sineshchekov et al., 2015) allow the passage of NO_3_^-^ and Cl^-^ ions in response to light. These channels also exhibit some conductivity for Br^-^ and I^-^ ions. The best-studied anion channelrhodopsins are GtACR1 and GtACR2 from the marine alga *Guillardia theta* (Cryptophyta, **Figure 4**) (Govorunova et al., 2015), with absorption maxima in the blue region of the light spectrum (515 nm and 470 nm, respectively) (**Figure 8**). Both have been shown to mediate the gating of Cl^-^ ions, and for GtACR1, the passage of NO_3_^-^ appears to be its natural function (Ohki et al., 2023).

Type-1 rhodopsins also include sensory rhodopsins (**Figure 7**), which are not involved in ion transport across membranes. Instead, sensory rhodopsins function as photoreceptors, signaling via a specific transmembrane or soluble cytoplasmic transducer protein that is functionally and structurally distinct from the animal G-proteins associated with type-2 rhodopsins (Bogomolni and Spudich, 1982; Krah et al., 1994; Hoff et al., 1997; Spudich, 2013). Sensory rhodopsin I (SRI) was the first light sensor discovered in a microorganism, specifically in the archaeon *Halobacterium* (Bogomolni and Spudich, 1982). It acts as a phototaxis receptor, modulating swimming behavior in response to light intensity gradients (Bogomolni and Spudich, 1982; Spudich, 2013).

Enzyme rhodopsins (**Figure 7**, **Figure 6**) combine the properties of light-sensitive retinal-binding domains with enzymatic activity and are found in eukaryotes such as fungi, green algae, and choanoflagellates (Avelar et al., 2014; Lamarche et al., 2017; Yoshida et al., 2017; Scheib et al., 2018; Tian et al., 2018a, 2018; Luck et al., 2019; Mukherjee et al., 2019; Ikuta et al., 2020; Sugiura et al., 2020; Broser, 2021; Tsunoda et al., 2021; Tian et al., 2022). Dimerization appears to be necessary for enzyme rhodopsins, and they lack both pumping and channel activities. Unusually, enzyme rhodopsins possess not seven, but eight transmembrane helices, with the extra helix (TM0) located at the N-terminus of the rhodopsin domain. Upon photon absorption, the retinal undergoes a conformational change, initiating light-regulated intramolecular signaling that subsequently controls an associated enzyme, thereby triggering downstream biochemical processes. The evolutionary origin of enzyme rhodopsins is thought to involve gene fusion events, where an ancestral rhodopsin gene was combined with genes encoding various enzymatic domains. There are essentially three families of enzyme rhodopsins: histidine kinase rhodopsins (HKRs), rhodopsin phosphodiesterases (RhoPDEs), and rhodopsin guanylyl cyclases (RhoGCs) (Luck et al., 2012; Avelar et al., 2014; Govorunova et al., 2017; Yoshida et al., 2017). In rhodopsin guanylyl cyclases and rhodopsin phosphodiesterases, light activates the enzyme activity, whereas in histidine kinase rhodopsins, light inhibits activity. These enzyme rhodopsins regulate the concentration of cyclic nucleotides by catalyzing their synthesis or degradation (Avelar et al., 2014; Yoshida et al., 2017; Scheib et al., 2018; Mukherjee et al., 2019; Ikuta et al., 2020; Sugiura et al., 2020). Cyclic nucleotides can act as secondary messengers in gene expression by activating transcription factors (Mcdonough and Rodriguez, 2011), and they are frequently involved in the regulation of cell type-specific gene expression during developmental processes (Shaulsky and Huang, 2005). In the alga *Ostreococcus* (Prasinodermophyta, **Figure 4**), light-dependent changes in cAMP levels affect the biosynthesis of cyclin A, which in turn interacts with retinoblastoma protein (RB) to regulate the cell cycle (Moulager et al., 2010). In *Chlamydomonas*, a correlation has also been demonstrated between rhodopsin activation and cAMP levels (Boonyareth et al., 2009).

Histidine kinase rhodopsins (HKRs) are light-regulated, ATP-dependent hybrid histidine kinase systems resembling two-component signaling pathways, some of which contain a guanylyl cyclase effector domain (Tian et al., 2018a). Light is detected by the rhodopsin (opsin, OPS) (**Figure 6**), which subsequently inhibits the activity of the histidine kinase domain (H kinase). In the absence of this inhibition, the histidine kinase domain undergoes autophosphorylation using a phosphoryl group from ATP. Following autophosphorylation, the kinase transfers the phosphoryl group to a response regulator domain (RR). The response regulator domain can interact with either attached or independent effector domains. In HKRs that possess an attached guanylyl cyclase domain (GC), the response regulator intramolecularly activates the GC domain, leading to the production of cGMP from GTP (**Figure 6**). The cGMP then serves as an effector molecule, triggering various cellular processes. A variety of other output responses can also be controlled through the response regulator domains (Galperin, 2006).

Rhodopsin phosphodiesterases (RhoPDEs) catalyze the hydrolysis of cAMP and cGMP to AMP and GMP, respectively, in a light-dependent manner (Watari et al., 2019; Ikuta et al., 2020; Sugiura et al., 2020). The N-terminal rhodopsin domain is linked to a C-terminal phosphodiesterase domain. Light-dependent isomerization of retinal induces a conformational change in the rhodopsin, which activates the catalytic domain of the phosphodiesterase. In this way, the degradation—and thus the intracellular concentrations—of the secondary messengers cAMP and cGMP can be dynamically regulated in response to light (Watari et al., 2019; Ikuta et al., 2020; Sugiura et al., 2020).

In rhodopsin guanylyl cyclases (RhoGCs), the N-terminal rhodopsin domain is directly linked to a C-terminal guanylyl cyclase (GC) domain (Avelar et al., 2014; Scheib et al., 2018; Tian et al., 2018a; Fischer et al., 2021). The first enzyme rhodopsin with confirmed enzymatic activity was a RhoGC identified in the fungus *Blastocladiella emersonii* (Saranak and Foster, 1997). Light-induced isomerization of retinal triggers a conformational change in the rhodopsin, thereby activating the guanylyl cyclase to produce cGMP from GTP (Avelar et al., 2014; Scheib et al., 2018; Tian et al., 2018a; Fischer et al., 2021).

There are also additional subclasses of rhodopsins, including heliorhodopsins, xanthorhodopsins, fungal rhodopsins, and viral rhodopsins (Rozenberg et al., 2021), which will not be discussed further here. **Figure 7** illustrates the main mechanisms of rhodopsins.

In algae, numerous light-controlled channels, pumps, and enzymes from the group of type-1 (microbial-type) rhodopsins have already been identified. Given the broad distribution of rhodopsins in algae and the large number of still poorly characterized algal species, it is likely that additional interesting rhodopsins will be discovered in the future.

### 5.3 Tetrapyrrole-based receptors

#### 5.3.1 Phytochromes

**Figure 9:**
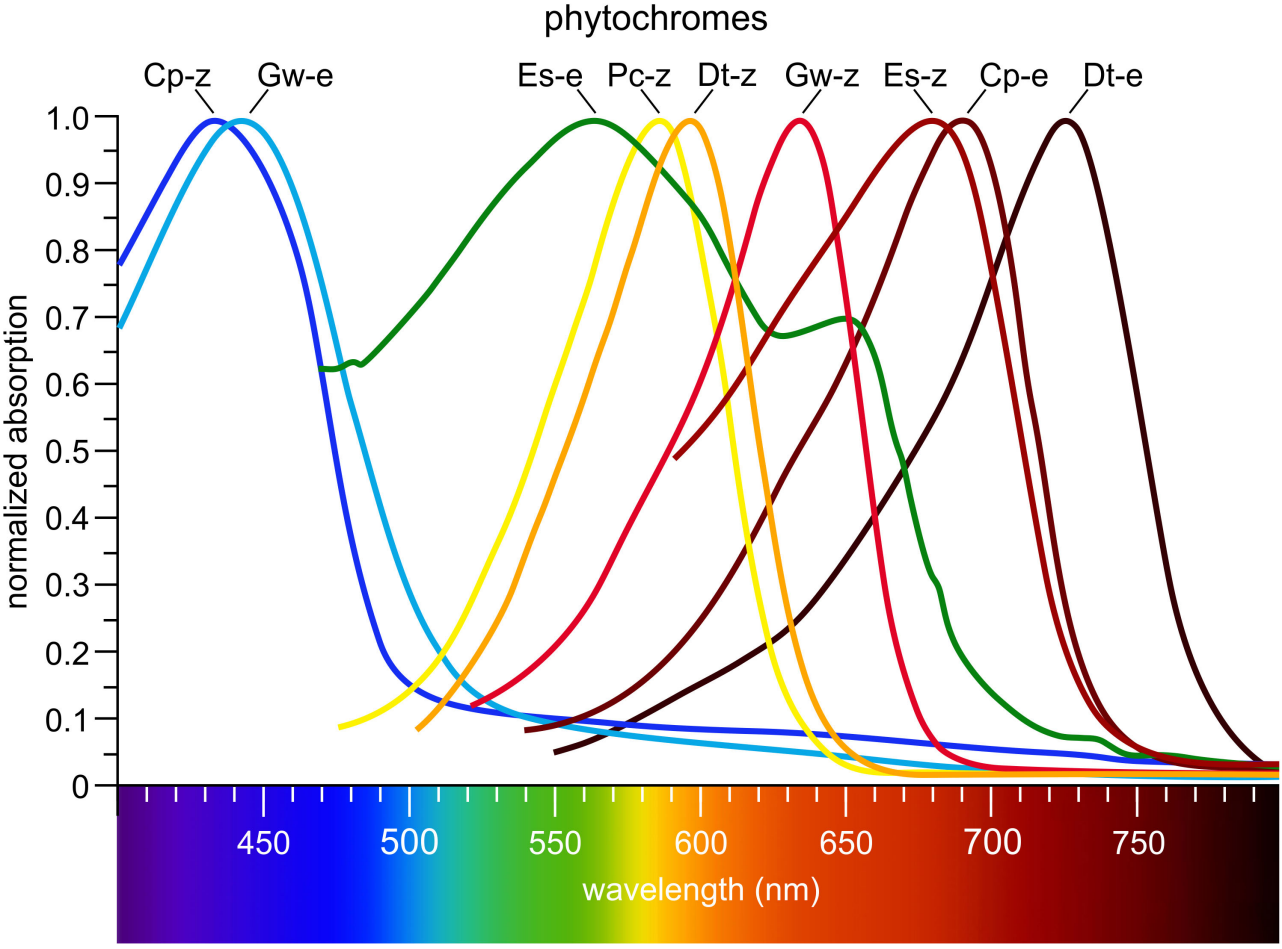
Algal phytochromes span the entire visible spectrum. Cp-z: *Cyanophora paradoxa* (Glaucophyta, **Figure 4**), 15Z CparGPS1; Gw-e: *Gloeochaete wittrockiana* (Glaucophyta), 15E GwitGPS1; Es-e: *Ectocarpus siliculosus* (Phaeophyta), 15E EsilPHL1; Pc-z: *Prasinoderma coloniale* (Prasinodermophyta), 15Z PcolPHY1; Dt-z: *Dolichomastix tenuilepis* (Chlorophyta), 15Z DtenPHY1; Gw-z: *Gloeochaete wittrockiana* (Glaucophyta), 15Z GwitGPS1; Es-z: *Ectocarpus siliculosus* (Phaeophyta), 15Z EsilPHL1; Cp-e: *Cyanophora paradoxa* (Glaucophyta), 15E CparGPS1; Dt-e: *Dolichomastix tenuilepis* (Chlorophyta), 15E DtenPHY1. Redrawn and modified after Rockwell et al. (2014). Phylogenetic positions are shown in **Figure 4**.

Phytochromes are highly conserved and widely distributed multi-domain proteins (**Figure 6**). These photoreceptors are present not only in algae, but also in plants, fungi, and bacteria (Ulijasz and Vierstra, 2011; Duanmu et al., 2014; Rockwell et al., 2014; Anders and Essen, 2015; Li et al., 2015a; Hughes and Winkler, 2024). For a long time, phytochromes were known only as red light photoreceptors, which could be reversibly switched between active and inactive states by a change in color from red to far-red light (Chen and Chory, 2011). However, over time, a wide range of phytochromes with different absorption spectra has been identified (**Figure 5**). In algae, phytochromes collectively cover the entire visible light spectrum (**Figure 9**) (Rockwell et al., 2014; Rockwell and Lagarias, 2020). As with all photoreceptors, but especially for phytochromes, an important ecological consideration is that the further the absorption maximum is shifted into the long-wavelength (red/far-red) range, the closer to the water surface the respective alga must live for that wavelength to be available (**Figure 2**) and thus detectable.

Phytochromes possess a characteristic cGMP phosphodiesterase/adenyl cyclase Fh1A (GAF) domain (**Figure 6**) that accommodates an open-chain tetrapyrrole chromophore. Unlike higher plants and mosses, which employ phytochromobilin (PΦB), green algal phytochromes utilize phycocyanobilin (PCB) (**Figure 6**) (Wu et al., 1997; Jorissen et al., 2002; Duanmu et al., 2013, 2017; Rockwell and Lagarias, 2017; Frascogna et al., 2023). The GAF domain mediates reversible photochromic transitions between two stable conformational states, most prominently the red light-absorbing Pr state and the far-red light-absorbing Pfr state. In most phytochromes, Pr represents the dark-adapted ground state, whereas Pfr is the photoactivated and often biologically active state (Franklin and Quail, 2010; Chen and Chory, 2011). This photoconversion enables organisms to discriminate between full sunlight and shaded conditions: in sunlight, phytochromes absorb red light and accumulate in the Pfr state, whereas in shade, where red light is depleted and far-red light is enriched, the spectral shift signals competition from neighboring photosynthetic organisms and promotes reversion to Pr either through far-red photoconversion or thermal relaxation in darkness. Beyond the red/far-red light pair, additional spectral pairs such as ultraviolet/blue, blue/green, or blue/orange have been described. A few phytochromes even adopt Pfr as the dark-adapted state, requiring far-red light for conversion back to Pr (Burgie and Vierstra, 2014; Rockwell and Lagarias, 2017). To date, however, no such Pfr-dark-adapted phytochromes (15E configuration) have been identified in algae. Classical plant and algal phytochromes instead adopt Pr (15Z configuration) in darkness and convert to Pfr upon red light absorption (Sharrock, 2008; Rockwell et al., 2014; Rockwell and Lagarias, 2020). Inverse dark-state configurations have thus far been reported only in certain bacterial phytochromes, most notably in the *Pseudomonas* phytochrome, which predominantly resides in Pfr in darkness (Yang et al., 2008; Rottwinkel et al., 2010; Rockwell and Lagarias, 2020; Huber et al., 2024).

In addition to the GAF domain, phytochromes contain several other domains (**Figure 6**) (Rockwell et al., 2006; Essen et al., 2008; Sharrock, 2008; Burgie and Vierstra, 2014). The phytochrome-specific PHY domain enhances the stability of chromophore binding, supports photoconversion by structurally stabilizing the GAF domain, and is essential for light perception and efficient switching between active and inactive states. A key structural feature of the PHY domain is the so-called tongue motif, a flexible loop that folds back onto the chromophore-binding pocket (Essen et al., 2008; Anders et al., 2013; Burgie and Vierstra, 2014). Upon photoconversion, the tongue undergoes pronounced secondary-structure transitions (typically from β-sheet to α-helix or vice versa), which represent one of the most prominent light-induced conformational changes in phytochromes. These rearrangements are thought to play a central role in transmitting the structural signal from the chromophore to the adjoining PHY and output domains, thereby linking local photochemistry to large-scale protein reorganization and signal propagation (Essen et al., 2008; Anders et al., 2013; Burgie and Vierstra, 2014). Together, these features establish the PHY domain as a central mediator between the chromophore-binding pocket and the downstream signaling machinery.

The PER-ARNT-SIM (PAS) domain, named after the proteins PER, ARNT, and SIM in which it was first described, is a versatile sensor and interaction module that occurs in many signaling proteins (Rockwell et al., 2006; Möglich et al., 2010; Burgie and Vierstra, 2014). In the N-terminal photosensor module of phytochromes, a PAS domain is tightly integrated into a compact, knotted architecture together with the GAF and PHY domains. This PAS–GAF–PHY unit harbors the bilin chromophore and mediates photoconversion (Rockwell et al., 2006; Burgie and Vierstra, 2014). By contrast, many eukaryotic phytochromes also possess one or more PAS repeats in their C-terminal region. These C-terminal PAS domains are structurally and functionally distinct from the N-terminal PAS domain and are thought to contribute primarily to dimerization, signal transmission, and interactions with downstream signaling partners (Essen et al., 2008; Sharrock, 2008; Burgie and Vierstra, 2014). The histidine kinase (H kinase) domain represents the signal transduction domain, often containing an autophosphorylating histidine residue, and ultimately transmits signals through phosphorylation to downstream targets such as transcription factors. Like other photoreceptors, phytochromes orchestrate a complex signaling network (Cheng et al., 2021; Han et al., 2024).

### 5.4 Hybrid receptors

#### 5.4.1 Neochromes

Neochromes are chimeric photoreceptors composed of six domains organized into two larger units (Nozue et al., 1998; Suetsugu et al., 2005; Kagawa and Suetsugu, 2007; Kong and Okajima, 2016; Parihar et al., 2016). The first unit comprises the three N-terminal domains and is structurally similar to phytochromes. The second unit consists of the three C-terminal domains and resembles phototropins. The three most N-terminal domains form a sensory module (**Figure 6**): these are the PER-ARNT-SIM (PAS) domain, the cGMP phosphodiesterase/adenyl cyclase Fh1A (GAF) domain, and a phytochrome-specific (PHY) domain, all typically found in phytochromes. The PAS domain mediates protein-protein interactions, contributes to protein dimerization, stabilizes the structure, and acts as a sensor for environmental stimuli such as physical or chemical signals. The GAF domain binds a phycocyanobilin chromophore, as in phytochromes, enabling the detection of (red) light signals. The PHY domain stabilizes the GAF domain, enhances chromophore binding, and supports photoconversion and effective light perception (Suetsugu et al., 2005; Kong and Okajima, 2016; Parihar et al., 2016).

The three most C-terminal domains form a phototropic region (**Figure 6**): these are two LOV domains (LOV1 and LOV2), which use flavin mononucleotide (FMN) as their chromophore, and a serine/threonine kinase (S/T kinase) domain (Shankar et al., 2022). The LOV domains absorb blue light; LOV1 also modulates interactions with LOV2 and other domains. Upon light absorption, LOV2 undergoes conformational changes that transmit signals to other parts of the protein, such as the S/T kinase domain. The S/T kinase domain catalyzes the phosphorylation of target proteins at serine or threonine residues, thereby regulating interactions with target proteins and initiating specific cellular responses (Suetsugu et al., 2005; Shankar et al., 2022).

Overall, neochromes are sensitive to red, far-red, and blue light (**Figure 5**), and they exhibit red/far-red reversibility. Neochromes were first identified in the green filamentous zygnematophycean alga *Mougeotia* (Charophyta, **Figure 4**), where they play an important role in regulating chloroplast movement in response to blue light stimuli (Suetsugu et al., 2005; Li et al., 2015a). Neochromes are found in zygnematalean algae and, outside of algae, in ferns and hornworts (Suetsugu et al., 2005; Li et al., 2014, 2015). Phylogenetic evidence indicates multiple independent origins of neochromes, with domain fusion as the underlying mechanism and horizontal gene transfer (HGT) shaping their distribution across lineages (Li et al., 2014, 2015, 2015; Li and Mathews, 2016).

### 5.5 Proteins with UV absorption

#### 5.5.1 UV-B photoreceptors

Ultraviolet-B (UV-B) radiation (280–315 nm) (**Figure 5**) is potentially harmful to all living organisms (Yin and Ulm, 2017). High doses of UV-B radiation can damage DNA and other macromolecules, induce the production of reactive oxygen species, and negatively affect cell viability (Brosché and Strid, 2003; Frohnmeyer and Staiger, 2003). However, UV-B radiation also serves as a signal that triggers various physiological responses. In algae, UV-B photoreceptors play a key role in sensing and responding to UV-B exposure. UVR8 (UV Resistance Locus 8) is the most important UV-B photoreceptor, found in both plants and algae (Rizzini et al., 2011; Tilbrook et al., 2013; Jenkins, 2014; Podolec et al., 2021; Depaepe et al., 2023). UVR8 exists as a homodimer that undergoes a conformational change upon UV-B exposure, resulting in dissociation into monomers, which in turn initiate downstream UV-B signaling events (Christie et al., 2012; Li et al., 2022; Depaepe et al., 2023). Before this conformational change, the homodimer is stabilized by interprotein interactions involving salt bridges, hydrogen bond networks, and hydrophobic clusters with entrapped water molecules (Li et al., 2022). The UV-B-dependent activation and dissociation of the dimer is unique because it does not require external chromophores (**Figure 6**); instead, tryptophan residues within the UVR8 polypeptide absorb the UV-B photons (Rai et al., 2019; Li et al., 2022). Upon activation, UVR8 monomers interact with signaling partners such as COP1 (CONSTITUTIVE PHOTOMORPHOGENIC 1), leading to the regulation of UV-B-responsive genes (Podolec et al., 2021). COP1, like UVR8, is highly conserved and is found not only in algae but also in plants and animals, including humans (Zhang et al., 2023). The UV-B-induced dissociation of the UVR8 dimer is reversible; the monomers spontaneously reassemble within hours, allowing the dimer to respond to UV-B again (Christie et al., 2012).

UV-B radiation detected by UVR8 also activates protective mechanisms, including the synthesis of UV-absorbing compounds such as carotenoids, mycosporine-like amino acids (MAAs), and antioxidant enzymes (Tilbrook et al., 2013; Singh et al., 2014; Ulm and Jenkins, 2015; Rosic, 2019; Groves and Franklin, 2025). In algae, these protective systems are often linked to the induction of genes required for the biosynthesis of these substances. DNA repair mechanisms can also be activated, such as photolyases, which repair DNA damage caused by UV radiation. Photolyases specifically catalyze the repair of UV-induced DNA lesions such as cyclobutane pyrimidine dimers (CPDs), which form when two adjacent pyrimidine bases (e.g., thymine or cytosine) in DNA become covalently linked. Pyrimidine-pyrimidone (6–4) photoproducts (6–4PPs), another type of UV-induced DNA damage involving covalent linkage between two pyrimidine bases, can also be repaired by photolyases. In addition to activating DNA repair systems, UV-B-activated photoreceptors mediate the protection of the photosynthetic machinery and regulate growth processes to prevent oxidative stress (Favory et al., 2009; Tilbrook et al., 2013; Jenkins, 2014; Müller-Xing et al., 2014; Allorent et al., 2016).

## 6 Algal genome and transcriptome data as sources of new light-sensitive proteins

Following the characterization of known algal light-sensing proteins, attention is now shifting toward the discovery of previously unrecognized photoreceptors, an endeavor greatly accelerated by the recent surge in algal genomics and transcriptomics. The primary resource for algal genomics is PhycoCosm (https://phycocosm.jgi.doe.gov), hosted by the Joint Genome Institute (JGI), and interconnected with plant genomes in JGI Phytozome (Goodstein et al., 2012) via the Embryophyta node. PhycoCosm contains over 200 algal genome projects from about 157 species, spanning the major algal lineages (**Figure 4**): Chlorophyta, Rhodophyta, Bacillariophyta, Charophyta, Haptophyta, Phaeophyta, Eustigmatophyta, and several smaller groups. This broad taxonomic coverage is crucial, as algae span highly diverse and evolutionarily complex—often bushy—branches of the tree of life.

Beyond genomics, PhycoCosm and related resources provide a wealth of multi-omics data, including transcriptomes, proteomes, and metabolomes. The expansion of such data has accelerated through initiatives such as the One Thousand Plant Transcriptomes Initiative (1KP) (Carpenter et al., 2019; One Thousand Plant Transcriptomes Initiative, 2019), the 10,000 Plant Genomes Project (10KP) (Cheng et al., 2018), and the Earth BioGenome Project (EBP), the latter of which aims to sequence and publicly release the genomes of all known eukaryotic species (Lewin et al., 2018; Lawniczak et al., 2022).

The availability of these comprehensive datasets allows for *in silico* identification of novel light-sensitive proteins and domains by sequence comparison and bioinformatics. This approach is not limited to algae but extends across photosynthetic and non-photosynthetic lineages. Newly identified genes can then be cloned, heterologously expressed, functionally characterized, and structurally modeled, revealing new properties and potential applications.

Earlier, *de novo* transcriptome sequencing of 127 algal species led to the identification of 61 channelrhodopsin homologs, which were subsequently expressed and physiologically characterized (Klapoetke et al., 2014). Among these were the well-known light-sensitive proteins Chronos and Chrimson. The ongoing exploration of these rapidly growing resources is expected to yield many more novel photoreceptors, further expanding the molecular toolkit for research and biotechnology.

## 7 Application of algal light-sensitive proteins in optogenetics

Optogenetics has revolutionized the ability to control the activity of genetically modified cells in living organisms, particularly within the field of neuroscience (Deisseroth, 2011; Zhang et al., 2011; Deisseroth, 2015; Hippler, 2017; Awasthi et al., 2020; Kojima et al., 2020; Emiliani et al., 2022; Tan et al., 2022; Zhang et al., 2022; Piatkevich and Boyden, 2023; Rindner and Lur, 2023; Busskamp et al., 2024; Levesque et al., 2024; Vlasova et al., 2024; Zhang et al., 2024b, 2024; He et al., 2025b; Pobozy et al., 2025; Zhou et al., 2025). This technique has been applied both for therapeutic purposes (Zarbin et al., 2013; Busskamp et al., 2024; Pobozy et al., 2025; Zhou et al., 2025) and to advance our understanding of brain circuitry (Deisseroth, 2011; Diester et al., 2011). Even optogenetic brain-computer interfaces are under development (Tang et al., 2024; Ahmed et al., 2025).

By employing light-sensitive proteins, researchers can manipulate neuronal activity with exceptional spatial and temporal precision. The fundamental principle behind optogenetic experiments is the use of genetically encoded optical actuators—light-controlled proteins that respond to specific wavelengths and intensities of light, transmitting signals to their targets and initiating precise biological responses.

The genes encoding these light-sensitive proteins originate from a diverse array of organisms, with microorganisms being the primary source. Among these, microalgae represent important contributors, but additional sources include archaea (e.g., halobacteria) that produce halorhodopsins and archaerhodopsins, as well as bacteria that contribute proteorhodopsins and various light-sensitive enzymes (Spudich et al., 2000; Zhang et al., 2011; Losi and Gärtner, 2012; Govorunova et al., 2017). Phytochromes and LOV domains are derived from cyanobacteria. The availability of a broad repertoire of light-controlled proteins and their respective genes is integral to the field of optogenetics.

While a wide range of light-sensitive proteins from different organisms have been adapted for optogenetics, the following section emphasizes channelrhodopsins. Among algal-derived photoreceptors, these have become the most widely adopted tools in optogenetics and remain the best-characterized in terms of structure, mechanism, and functional versatility.

A hallmark of optogenetic research is the use of channelrhodopsins (Bamann et al., 2010; Hou et al., 2011; Lin, 2011; Prigge et al., 2012; Hegemann and Nagel, 2013; Guru et al., 2015; Mcisaac et al., 2015; Schneider et al., 2015; Wietek and Prigge, 2016; Govorunova et al., 2017; Losi et al., 2018; Coli et al., 2024; Pobozy et al., 2025; Wang et al., 2025), a class of light-sensitive proteins from algae that play a pivotal role in manipulating neuronal activity. The discovery of channelrhodopsins ChR1 and ChR2 in *Chlamydomonas reinhardtii* marked the inception of the field of optogenetics (Nagel et al., 2002, 2003). Since then, numerous additional channelrhodopsins have been identified in various chlorophytes, thereby expanding the potential of optogenetic tools. The development of mutated channelrhodopsin variants has driven significant technological progress. These engineered variants possess altered functional properties, such as modified channel closing kinetics or spectral sensitivities shifted toward blue or red light, which are crucial for broadening the versatility of optogenetic applications (Berndt et al., 2009; Lin et al., 2009; Kleinlogel et al., 2011; Lin et al., 2013; Klapoetke et al., 2014). Notably, ChR2 from *C. reinhardtii* has become the gold standard in optogenetics, serving as the prototype for further variants designed to optimize photocurrent amplitude, response speed, and light sensitivity (Boyden et al., 2005; Nagel et al., 2005; Lin et al., 2009; Gunaydin et al., 2010; Berndt et al., 2011). Extensive color tuning, which modifies the spectral sensitivity of these proteins, has opened new avenues for experimental design (**Figure 8**) (Mattis et al., 2011; Chuong et al., 2014; Klapoetke et al., 2014; Olofsson et al., 2015; Wietek and Prigge, 2016).

The development of optogenetic tools typically follows a bottom-up approach, whereby novel channelrhodopsins are first identified and characterized before being engineered to meet specific experimental requirements. In neurobiology, for instance, the expression of channelrhodopsins under the control of cell type- and species-specific promoters allows for the selective activation of defined neuronal populations, ensuring precise spatial and temporal control (Boyden et al., 2005; Fenno et al., 2011; Zhang et al., 2011; Deisseroth, 2015). Optogenetics thus enables exceptionally accurate and specific manipulation of neuronal activity.

Numerous engineered channelrhodopsin variants have been developed to address a wide range of research needs (**Figure 8**). These include various ChR2 variants from *C. reinhardtii*, such as ChR2-H134R (Nagel et al., 2005; Mattis et al., 2011), ChR2(E123T/T159C) (Berndt et al., 2011), ChD, ChF, ChEF (Lin et al., 2009, 2013), ChIEF (Lin et al., 2013), ChR2E/T, and ChETA (Gunaydin et al., 2010). From a ChR2-homologous channelrhodopsin from *Scherffelia dubia* (Chlorodendrophyceae), SdChR, which produces larger photocurrents than ChR2, further valuable optogenetic tools have been developed. One such derivative is SdChR(E154A), known as CheRiff (Hochbaum et al., 2014; Venkatachalam and Cohen, 2014). Among all channelrhodopsins, the ChR2-homologous channelrhodopsin TsChR from *Tetraselmis striata* (Chlorodendrophyceae) is the most blue-shifted, absorbing at the shortest wavelength (~436 nm), but it generates only weak photocurrents (Klapoetke et al., 2014). The engineered variant eTsChR, based on TsChR, exhibits improved trafficking and robust neuronal spiking (Farhi et al., 2019). Another ChR2-homologous channelrhodopsin, CoChR from *Chloromonas oogama* (Chlamydomonadaceae), demonstrates high light-driven photocurrents but slow channel kinetics. Here, optimized variants with enhanced light sensitivity and maintained high photocurrent amplitudes have been engineered, including CoChR-LC (CoChR-L112C), CoChR-3M (CoChR-H94E/L112C/K264T), and CoChR(H94E/E103A) (Ganjawala et al., 2019; Bi et al., 2022).

New channelrhodopsin variants have also been created through the construction of fusions and chimeras from two or more sequences (**Figure 8**). Notably, chimeras derived from ChR1 (*C. reinhardtii*) and VChR1 (*V. carteri*)—which contain no ChR2 sequence—such as C1V1TT (Yizhar et al., 2011) and mVChR1 (Tomita et al., 2014), are sensitive to green light. The optimized variant ComV1(ex3mV1Co) (Tomita et al., 2014; Watanabe et al., 2021) exhibits enhanced sensitivity under daylight conditions. White-opsin is a fusion of three distinct opsins—ChR2, C1V1TT, and ReaChR—each responsive to different regions of the visible spectrum (blue, green, and red wavelengths, respectively) (Batabyal et al., 2015), thereby conferring broad-spectrum sensitivity.

Red-shifted channelrhodopsins are of particular importance for optogenetics because they respond to longer wavelengths of light, enabling deeper tissue penetration and minimizing phototoxicity (**Figure 8**). VChR1 from *V. carteri*, discovered after ChR2, was noteworthy for its more than 50-nm red shift compared to ChR2. Subsequently, an even more red-shifted channelrhodopsin was identified: CnChR from *C. noctigama* (Chlamydomonadaceae), commonly known as Chrimson (Klapoetke et al., 2014). Additional red-shifted variants, such as ReaChR (red-activatable channelrhodopsin), were engineered from VChR1. ReaChR’s activation spectrum peaks at ~530 nm with short light pulses, but longer pulses can further shift the peak to ~580 nm (Inagaki et al., 2014). The variant bReaChES is a faster red-shifted channelrhodopsin, generated by Glu123Ser substitution and replacement of the N-terminal residues in ReaChR with those of ChR2 (Kim et al., 2016). The yellow-orange-peaked Chrimson has served as the basis for a family of derivatives: CsChrimson, f-Chrimson (Chrimson Y261F/S267M), vf-Chrimson (Chrimson K176R/Y261F/S267M), ChrimsonR (Chrimson K176R), and ChrimsonSA (Klapoetke et al., 2014; Oda et al., 2018; Gupta et al., 2019).

Another highly sought-after property of channelrhodopsins is fast kinetics. The most notable example is ShChR from *Stigeoclonium helveticum* (Chaetophoraceae), usually referred to as Chronos (Klapoetke et al., 2014). While faster kinetics typically result in reduced light sensitivity, Chronos is the fastest channelrhodopsin identified and still maintains relatively high light sensitivity (Mardinly et al., 2018). Its absorption maximum lies in the blue-green range (~500 nm). Chronos derivatives include ChroME, ChroME2.0, ChroME2f, ChroME2s, ST-ChroME, and ChrMD (Sridharan et al., 2022). In neurobiology, Chronos and its mutants enable rapid neuronal activation with considerable light sensitivity. Combining Chronos and Chrimson enables two-color activation of neural spiking and downstream synaptic transmission in neurobiological experiments (Klapoetke et al., 2014).

Bistable channelrhodopsins, also known as step-function opsins (SFOs), represent a distinct functional class. They sustain prolonged photocurrent activation even after the light stimulus has ceased and exhibit sensitivities several orders of magnitude higher than those of fast channelrhodopsins (Berndt et al., 2009; Guru et al., 2015). ChR2(C128A) and ChR2(C128S) were the first SFOs, generated by single amino acid mutations at C128 in the ChR2 sequence, which extend the open-state lifetime to tens of seconds. ChR2-C128S can be turned on with blue light (~450 nm) and turned off with yellow light (~550 nm). The double mutant ChR2(C128S/D156A), known as stabilized step-function opsin (SSFO), maintains stable photocurrents for several minutes (Yizhar et al., 2011; Guru et al., 2015).

Another ChR2 mutant, ChR2(L132C), exhibits a sixfold increase in calcium ion permeability (Kleinlogel et al., 2011), and is termed CatCh (Calcium transporting Channelrhodopsin). In animal models, CatCh enables neuronal activation at much lower light intensities, reducing the risk of phototoxicity. Calcium affinity was further enhanced by targeted mutagenesis at various positions, generating CapChRs (Calcium-permeable Channelrhodopsins) that exhibit reduced sodium and proton conductance alongside markedly improved Ca^2+^ permeation at negative voltage and low extracellular Ca^2+^ concentrations (Fernandez Lahore et al., 2022).

Mutation of E90 in ChR2 to the positively charged amino acid arginine can even convert ChR2 into a light-gated chloride channel (ChloC) (Wietek et al., 2014). Ion selectivity has also been altered in the engineered channelrhodopsin PsCatCh2.0, derived from the highly blue-shifted *Platymonas subcordiformis* (Chlorodendraceae) PsChR (**Figure 8**) through an L115C mutation and further modifications (Govorunova et al., 2013; Chen et al., 2022; Pobozy et al., 2025). Due to its low proton permeability, which minimizes perturbations to cellular pH, this variant is well suited for clinical applications where acidification is undesirable.

Synthetic opsins such as MCO1 (Multi-Characteristic Opsin 1) and MCO-010 have also been developed (Wright et al., 2017a, 2017). These opsins exhibit broad spectral sensitivity, high light sensitivity, and fast kinetics, and are currently being tested for the treatment of retinal degenerative diseases such as retinitis pigmentosa and Stargardt disease (Mohanty et al., 2025). Additionally, machine learning-guided engineering approaches (Bedbrook et al., 2019) have resulted in new channelrhodopsins such as ChRger1, ChRger2, and ChRger3. AI-based methods are expected to produce further variants in the near future.

In the context of neurological disorders, optogenetic approaches are being explored for the treatment of Parkinson’s disease, epilepsy, and depression. Researchers use light to modulate specific neuron populations, potentially correcting aberrant neural activity (Deisseroth, 2015; Kianianmomeni and Hallmann, 2015a). Optogenetic strategies are also being investigated for vision restoration in conditions such as retinitis pigmentosa, by rendering retinal cells light-sensitive (Sahel et al., 2021; Pobozy et al., 2025).

Similarly, optogenetic strategies are under investigation for cochlear implants. Instead of electrical impulses, light stimulation could provide more precise targeting of auditory neurons, potentially improving sound resolution and speech recognition in noisy environments (Wrobel et al., 2018; Huet et al., 2024). There are also optogenetic applications in oral and craniofacial fields (Zhang et al., 2024c).

Furthermore, in cardiac medicine, optogenetics is being studied as a method to control heart rhythms by targeting cardiac cells with light, offering potential treatments for arrhythmias (Bruegmann et al., 2018). Optogenetic techniques are also being researched to modulate pain pathways and may offer alternatives to conventional painkillers (Iyer et al., 2014; Li and Ji, 2024). Moreover, pancreatic beta cells have been engineered using optogenetic techniques to regulate insulin release in response to light. This approach holds potential as a strategy for managing type 1 diabetes by enabling precise, non-invasive control of insulin secretion (Shao et al., 2017).

Overall, optogenetics remains a vital tool for investigating complex biological processes and is facilitating the development of novel medical therapies. Numerous studies and reviews provide detailed descriptions of this foundational research (Fenno et al., 2011; Hegemann and Nagel, 2013; Tischer and Weiner, 2014; Deisseroth, 2015; Gao, 2015; Guru et al., 2015; Kianianmomeni and Hallmann, 2015a).

Looking forward, the integration of different light-sensitive proteins for multicolor optogenetics is expected to become increasingly important. Addressing challenges such as spectral overlap, crosstalk, and interference between different opsins or light sources will be crucial. Optogenetic tools will also continue to aid in mapping entire neural circuits and exploring complex brain functions. Applications in non-neuronal cells (e.g., muscle cells, immune cells, or engineered tissues) should be further expanded. Light could be used to control cell growth, differentiation, or migration, potentially enabling tissue-specific therapies where cells are engineered for optogenetic control. Finally, there is also a need for improved light delivery systems and more precise control of light exposure.

## 8 Conclusion and perspectives

Algae have evolved a remarkable array of photoreceptors that allow them to sense and adapt to an extraordinary diversity of light environments. This diversity is not only a product of their evolutionary history—marked by endosymbiotic events, gene duplications, and domain fusions—but also a reflection of the tremendous variety of ecological niches they occupy, from sunlit surface waters to deep oceans, shaded terrestrial environments, and extreme habitats.

The main classes of algal photoreceptors—flavin-based (phototropins, cryptochromes, aureochromes, BLUF proteins), retinal-based (rhodopsins), tetrapyrrole-based (phytochromes), hybrid (neochromes), and UV-B receptors (UVR8)—collectively cover the entire spectrum from ultraviolet to far-red light. These receptors underpin a broad range of physiological processes, including photosynthesis, photoprotection, phototaxis, circadian rhythms, development, sexual cycles, and pigment synthesis. The interplay between different photoreceptors and signaling pathways enables algae to finely tune their responses to fluctuating light conditions and to integrate light signals with other environmental cues.

The rapid expansion of algal genomics and transcriptomics, supported by large-scale sequencing initiatives, is accelerating the discovery of new light-sensitive proteins. Bioinformatic and functional characterization of these photoreceptors is expanding our understanding of algal biology and providing a rich source of novel molecular tools.

A particularly transformative application of algal photoreceptors has been in optogenetics. Channelrhodopsins and other light-sensitive proteins from algae have revolutionized neuroscience by enabling precise, light-controlled manipulation of neuronal and other cell activities. The ongoing engineering of channelrhodopsins for altered spectral sensitivity, kinetics, and ion selectivity continues to enhance the versatility of optogenetic techniques.

Future directions include:

– Continued discovery and functional analysis of novel photoreceptors from diverse algal groups, facilitated by advances in sequencing, bioinformatics, and synthetic biology.
– Integration of multiple light-sensitive proteins for complex, multicolor optogenetic control of cellular processes.
– Expansion of optogenetic applications beyond neuroscience, including cardiac, endocrine, and immunological systems, as well as in plant and algal biotechnology.
– Development of improved light delivery systems and opsin variants for use in tissues with limited light penetration.
– Use of machine learning and protein engineering to design custom photoreceptors with tailored properties for specific research and therapeutic needs.

The study of algal photoreceptors not only enriches our understanding of how life adapts to light but also fuels technological innovation in biology and medicine. As the molecular diversity of algal photoreceptors continues to unfold, these proteins will remain at the forefront of both fundamental discovery and applied science.
